# Spatial-temporal recurrence patterns of grade 4 glioma using deep learning integrated multiparametric MRI and molecular pathology

**DOI:** 10.1038/s41698-026-01557-0

**Published:** 2026-06-13

**Authors:** Marianne H. Hannisdal, Arvid Lundervold, Dorota Goplen, Jorunn Brekke, Mohummad A. Rahman, Petter Brandal, Tora S. Solheim, Stein Atle Lie, Nello Blaser, Martha Chekenya

**Affiliations:** 1 Cancer Clinic, Haukeland University Hospital, Bergen, Norway; 2 Department of Biomedicine, University of Bergen, Bergen, Norway; 3 Mohn Medical Imaging and Visualization Centre, Department of Radiology, Haukeland University Hospital, Bergen, Norway; 4 Department of Oncology, The Norwegian Radium Hospital, Oslo University Hospital, Oslo, Norway; 5 Institute of Clinical Medicine, University of Oslo, Oslo, Norway; 6 Institute for Cancer Genetics and Informatics, Oslo University Hospital, Oslo, Norway; 7 Cancer Clinic, St. Olavs Hospital, Trondheim University Hospital, Trondheim, Norway; 8 Department of Clinical and Molecular Medicine, Faculty of Medicine and Health Sciences, Norwegian University of Science and Technology, Trondheim, Norway; 9 Department of Clinical Dentistry, University of Bergen, Bergen, Norway; 10 Faculty of Science and Technology, University of Bergen, Bergen, Norway

**Keywords:** Biomarkers, Cancer, Oncology

## Abstract

Grade 4 glioma is inherently lethal due to inevitable recurrence. Current radiotherapy guidelines recommend uniform target volume margins, disregarding molecular and clinical variables. We hypothesized that spatiotemporal recurrence may be predicted by molecular and phenotypic features present already at diagnosis. We analyzed 390 paired MRIs of grade 4 gliomas from Norwegian multi-centers and TCIA (2015–2025). Deep learning segmentation identified contrast-enhancing (CEcore) and non-enhancing (NE) tumor compartments and afflicted anatomical regions. We quantified primary CEcore/NE volume ratio, anatomical tumor trajectories, and spatiotemporal progression using Hausdorff-95—analyzed in relation to survival, *MGMT*, *IDH*, age, extent of resection, sex, and anatomical location, using machine learning and Cox regression. Primary tumor composition was independently prognostic: CEcore/NE volume ratio ≤0.324 predicted improved overall survival (adjusted HR = 0.56, 95% CI 0.37–0.84, *p* = 0.006), independent of age, *MGMT*-status, and individual compartments. Within *IDH*-wildtype, *MGMT*-unmethylated patients, low volume ratio ≤0.324 demonstrated a 4.3 months survival benefit (median 17.6 vs 13.3 months, *p* = 0.0209). Longer time to progression correlated with increased HD95 distances (*p* < 0.03). Tumors originating in occipital lobe had highest propensity to migrate to new sites (57.1%) and the shortest time to progression (adjusted HR = 1.90, *p* = 0.026). These findings support molecular-demographic-anatomical risk stratification that may inform personalized margin-determination in radiotherapy planning.

## Introduction

Gliomas are common primary brain tumors in adults^1^. Amongst these, a subset harboring wild-type isocitrate dehydrogenase (*IDH*-wt.) gene, glioblastoma (GBM), represents the most frequent highest malignancy grade 4. They are most intractable due to diffuse infiltrative invasion, extensive genetic heterogeneity and cellular plasticity that render them treatment resistant. Standard treatment includes surgical resection which, depending on location may excise the majority of the contrast-enhancing tumor within safe acceptable margins^2^. However, diffusely infiltrative cells that migrate along neuronal pathways, white matter tracts, perivascular- and leptomeningeal spaces, evade surgical removal and form seeds for future recurrence. Post-surgical management involves fractionated radiotherapy (RT) with concurrent temozolomide (TMZ) chemotherapy, to which diffusely migrating cells are often refractory. Tumor recurrence is therefore inevitable, often within 2 cm of the resection cavity^3^, resulting in a dismal survival prognosis of approximately 9–15 months^1,4^. Patients’ prognosis and response to treatment differ depending on age at diagnosis, Karnofsky Performance Score (KPS), extent of surgical resection (EOR), and promoter methylation status of the DNA repair gene, O6-methylguanine-DNA methyltransferase (*MGMT*)^1,2,4–6^.

Although therapeutic benefit of RT is well-established, survival endpoints are dependent on accurate target delineation^7^. The European Society for Radiotherapy and Oncology (ESTRO) and European Association of Neuro-Oncology (EANO) guidelines recommend a margin of 10–15 mm beyond the visible tumor^8^. Although not specifying molecular or clinical stratification variables, the margin recommendation is informed by recurrence-pattern studies, several of which have demonstrated systematic differences in glioblastoma recurrence patterns associated with *MGMT* promoter methylation^8–11^. There remains a knowledge gap regarding how molecular markers may inform spatial risk assessment and how this should be accounted for when defining radiation target volumes. The interplay between recurrence patterns and molecular markers, anatomical locations, and patient demographics that ultimately influence survival outcomes is not clearly elucidated.

In this study, we integrated deep learning segmentation of tumor compartments, volumetrics, and tumor location within anatomical regions, with molecular genetics and demographic factors. We investigated features that predicted when and where the recurrent tumor localized, and its recurrence pattern relative to the composition and location of the primary tumor at diagnosis. We then elucidated how these salient features impacted survival outcomes. By characterizing spatiotemporal recurrence patterns through paired imaging analysis, we aimed to provide biological insights that may inform treatment planning and monitoring for future recurrences.

## Results

The study population comprised 195 patients with recurrent WHO grade 4 gliomas, median age was 58 years (range 24–83). Their demographics, molecular markers, EOR and tumor location at primary diagnosis are summarized in Table 1. The median time to progression (TTP) was 8.8 months (interquartile range [IQR] 6.1–13.1), range 1–55.5. Median overall survival (OS) was 17.8 months (IQR: 11.7–24.6 months, range: 2.4–91.9 months). Of the *n* = 195, 144 (73.8%) patients survived 1 year, 54 (27%) survived 2 years; and 3 (2%) were long-term survivors, living 5 years. Patients received diverse, physician´s choice treatments for their recurrent disease.

**Table 1:** Patient demographics, molecular characteristics, treatment, and survival outcomes for the full study cohort (*n* = 195)

Characteristics		Time to progression	Overall survival	Non-adjusted analysis		Adjusted analysis	
	*n* (%)	Median months (IQR)		HR (95 CI)	*p*-value	HR (95 CI)	*p*-value
**Demographics**							
Age	195 (100%)	8.8 (6.1–13.1)	17.8 (11.8–24.9)	1.35^a^ (1.17–1.57)	**<0.001**	1.59^a^ (1.31–1.93)	**<0.001**
Sex-no. (%)							
Male	130 (66.7%)	8.8 (6.5–13.7)	17.7 (11.7–24.8)	1	-	1	-
Female	65 (33.3%)	8.7 (5.3-12.5)	17.8 (12.7–24.4)	0.95 (0.71–1.28)	0.748	0.89 (0.64–1.26)	0.518
**Molecular markers**							
*MGMT*-promoter status							
Unmethylated^b^	127 (65.1%)	8.6 (6.1–11.9)	16.7 (11.5–22.1)	1	-	1	-
Methylated	51 (26.1%)	10.5 (6.6–18.3)	22.2 (14.5–31.2)	0.54 (0.39–0.77)	**<0.001**	0.41 (0.28–0.60)	**<0.001**
Unknown	17 (8.7%)	12.0 (7.2–23.8)	17.9 (13.7-22.8)	1.15 (0.69–1.93)	0.572	1.23 (0.72–2.11)	0.443
*IDH*-status							
Wildtype, glioblastoma	176 (90.2%)	8.7 (6.1–12.5)	17.5 (11.7–23.2)	1	-	1	-
Mutated, astrocytoma	11 (5.6%)	14.0 (5.9–16.9)	28.9 (27.3–33.9)	0.46 (0.25–0.86)	**0.014**	0.69 (0.35–1.38)	0.294
Unknown/Inconclusive	8 (4.1%)	12.4 (7.2–23.8)	22.0 (13.7–27.5)	0.56 (0.26–1.20)	0.136	0.81 (0.34–1.96)	0.652
Extent of surgical resection							
Gross total resection	71 (47.9%^c^)	9.5 (6.6–12.3)	17.7 (12.5–25.9)	1	-	1	-
Subtotal resection	52 (35.1%^c^)	7.4 (6.0–12.7)	15.2 (8.5–25.1)	1.13 (0.82–1.56)	0.443	1.42 (0.99–2.03)	0.056
Biopsy	25 (16.9%^c^)	10.0 (6.5-13.8)	17.7 (12.1–24.1)	0.95 (0.63-1.46)	0.832	1.22 (0.75–2.00)	0.422
Unknown	47	8.7 (5.6–13.2)	17.9 (12.6–23.9)	0.98 (0.71–1.37)	0.922	0.68 (0.46-1.03)	0.070
Primary tumor location							
Frontal	43 (22.0%)	10.0 (6.1–16.3)	21.8 (13.7–27.8)	0.87 (0.62–1.23)	0.428	1.11 (0.75–1.13)	0.594
Fronto-parietal junction	15 (7.7%)	10.0 (6.1–15.3)	26.0 (20.1–28.9)	0.73 (0.43–1.25)	0.254	0.92 (0.51–1.68)	0.794
Parietal	34 (17.4%)	8.7 (6.5–11.9)	17.0 (13.2–22.5)	1.17 (0.81–1.70)	0.406	0.85 (0.56–1.28)	0.436
Temporal	66 (33.8%)	9.7 (6.6–13.3)	17.5 (12.0–23.2)	0.89 (0.66–1.21)	0.464	0.81 (0.59–1.13)	0.217
Temporo-parietal junction	11 (5.7%)	6.6 (4.2–14.8)	17.9 (11.1–20.6)	1.09 (0.59–2.04)	0.779	1.25 (0.56–2.81)	0.583
Occipital	14 (7.1%)	7.6 (6.1–9.3)	15.6 (11.7–19.7)	1.27 (0.74–2.20)	0.387	1.35 (0.76–2.9)	0.311
Brainstem/cerebellum	3 (1.5%)	6.0 (4.9–10.5)	12.1 (6.1–35.6)	1.03 (0.33–3.24)	0.959	0.93 (0.29–3.01)	0.909
Midline/bilateral	9 (4.6%)	6.4 (4.5–9.6)	11.3 (6.7–12.5)	3.69 (1.86–7.32)	**<0.001**	3.77 (1.80–7.89)	**<0.001**
Recurrence							
Within same lobe	134 (68.7%)	8.6 (6.1–13.1)	17.6 (11.9–22.8)	1	-	1	-
Different lobe	61 (31.3%)	9.4 (6.6–13.4)	17.9 (12.0–26.6)	0.84 (0.61–1.14)	0.268	0.79 (0.56–1.13)	0.195

Patient and tumor characteristics are presented alongside time to progression, overall survival, and Cox proportional hazards regression for overall survival. For each categorical variable, the first listed level serves as the reference category for the regression analyses (denoted by HR = 1). Time to progression and overall survival are reported as median months with interquartile range (IQR). Hazard ratios (HRs) with 95% confidence intervals (CIs) are reported for both non-adjusted (univariable) and adjusted (multivariable) Cox models; the adjusted model included age, sex, O⁶-methylguanine-DNA methyltransferase (MGMT) promoter methylation status, isocitrate dehydrogenase (IDH) mutation status, extent of surgical resection (EOR), contrast-enhancing core (CEcore) volume, and non-enhancing (NE) tumor volume as covariates.

^a^Hazard ratio expressed per standard deviation increase of 11.65 years.

^b^The high frequency of unmethylated MGMT reflects the inclusion criteria of the NCT03643549 trial, which enrolled only MGMT-unmethylated patients.

^c^Percentages calculated from the *n* = 148 patients with known EOR status. Statistical significance is denoted in bold (*p* < 0.05).

### Machine learning identifies key predictors of tumor progression and survival

**Fig. 1:**
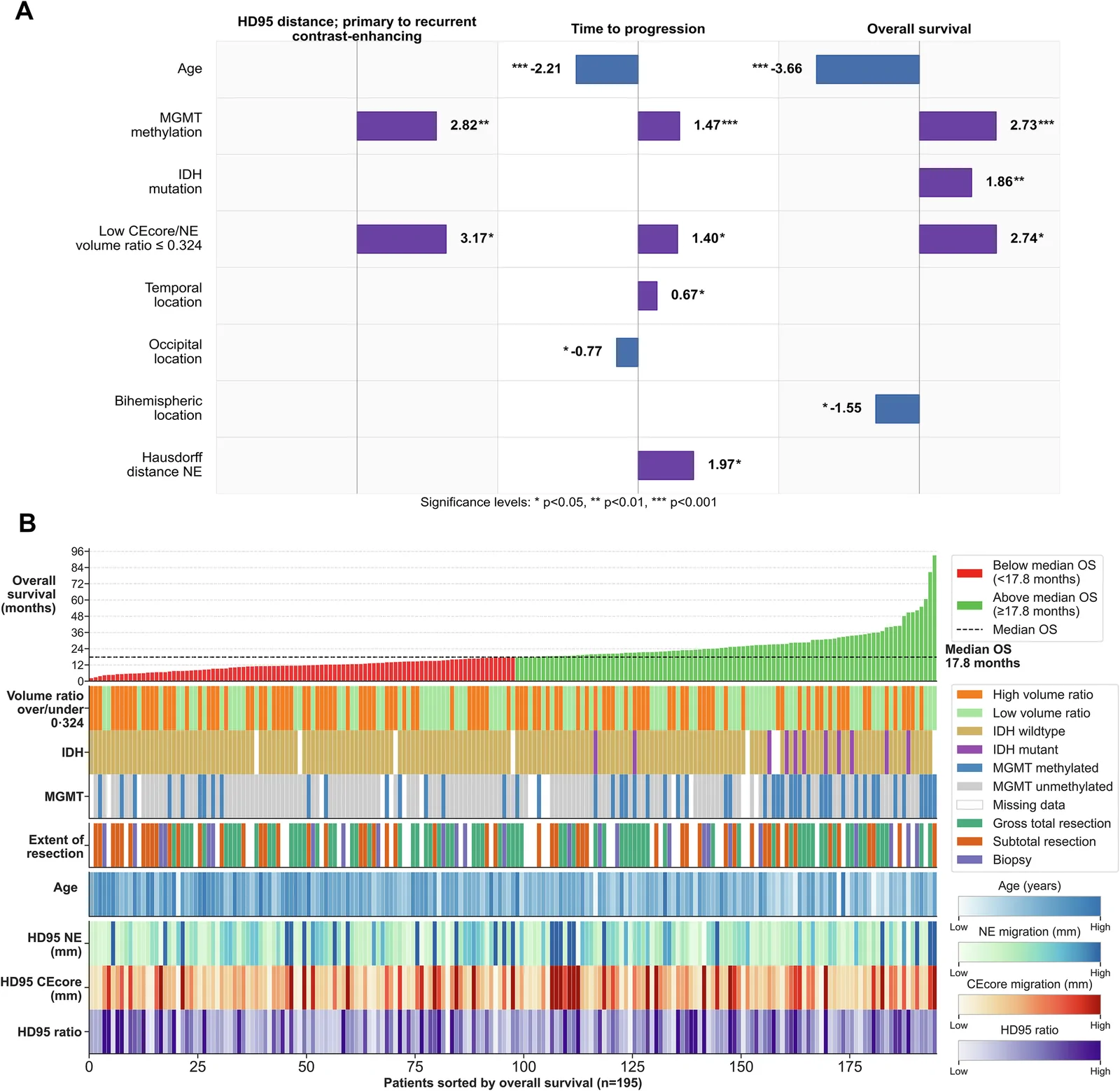
Machine learning feature importance across outcome measures and patient-level visualization of clinical, molecular, and imaging characteristics sorted by overall survival. **A** Machine learning model feature importance for prediction of three outcomes: the 95th percentile Hausdorff distance (HD95) between the primary and recurrent contrast-enhancing (CE) tumor compartments; time to progression; and overall survival. Coefficients were derived from Huber Regressor models selected via a PyCaret pipeline. Bar length represents the magnitude of the standardized regression coefficient, with purple bars indicating positive coefficients and blue bars indicating negative coefficients. Numerical coefficient values are shown adjacent to each bar. Statistical significance is denoted by asterisks (*p < 0.05, **p < 0.01, ***p < 0.001). Empty cells indicate that the feature was not retained as statistically significant for the corresponding outcome. Features evaluated include age at diagnosis, O⁶-methylguanine-DNA methyltransferase (MGMT) promoter methylation status, isocitrate dehydrogenase (IDH) mutation status, low contrast-enhancing including core to non-enhancing (CEcore/NE) volume ratio (≤0.324), temporal lobe primary location, occipital lobe primary location, bihemispheric primary location, and the HD95 of the non-enhancing (NE) compartment. **B** Patient-level visualization of the full cohort (*n* = 195), with patients arrayed along the horizontal axis in order of increasing overall survival (left to right). The top track displays overall survival in months as vertical bars, colored red for patients with overall survival below the cohort median of 17.8 months and green for patients at or above the median; the horizontal black dashed line marks the median. Below the survival plot, stacked color-coded tracks display, for each patient: the primary CEcore/NE volume ratio relative to the 0.324 cut-off (orange, high ratio; light green, low ratio); IDH mutation status (tan, wildtype; purple, mutant); MGMT promoter methylation status (blue, methylated; gray, unmethylated; white, missing data); extent of surgical resection (green, gross total resection; orange, subtotal resection; purple, biopsy); age at diagnosis (blue gradient, light to dark indicating young to old); HD95 of the NE compartment (green-to-blue gradient, light to dark indicating short to long); HD95 of the CEcore (red gradient, light to dark indicating short to long); and the HD95 migration ratio (HD95 of the CEcore divided by HD95 of the NE compartment; purple gradient, light to dark indicating low to high).

To systematically identify the most significant predictors of tumor behavior and outcomes from our dataset, we first employed machine learning (ML) to evaluate relative feature importance for the primary outcome: the Hausdorff-95 (HD95) recurrence distance of the contrast-enhancing tumor compartment including necrotic core (CEcore) and its impact on time to progression, and overall survival. Significant features are summarized in Fig. 1A.

For HD95 distances, primary tumor composition was a key determinant with CEcore/non-enhancing (NE) volume ratios below 0.324 at primary diagnosis predicting more distal CEcore recurrence (coefficient = 3.172, *p* = 0.046). Furthermore, methylated *MGMT*-promoter also predicted prolonged recurrence distance (coefficient = 2.821, *p* = 0.0027), indicating methylated tumors migrate spatially further.

For survival outcomes, our models confirmed established predictors of age, MGMT-, and IDH- status. Low CEcore/NE volume ratio predicted improved OS (coefficient = 2.74, *p* = 0.014) as well as prolonged time to progression (coefficient = 1.40, *p* < 0.011), and notably more distal tumor recurrence (coefficient 3.17, *p* = 0.046). Regarding anatomical locations, temporal lobe location predicted prolonged time to progression (coefficient = 0.67, *p* = 0.021), while occipital lobe location predicted shorter time to progression (coefficient = −0.77, *p* = 0.034). As expected, bi-hemispheric involvement predicted shorter OS (coefficient = −1.553, *p* = 0.016). ML model performance metrics are detailed in Supplementary Table 1.

We next sought to visualize how clinical features clustered with survival outcomes in the cohort (Fig. 1B), demonstrating clear patterns of favorable prognostic factors clustering among longer survivors. Interestingly, lower CEcore/NE ratios (≤0.324) and greater recurrence distances of CEcore consistently segregated with established positive prognostic indicators such as younger age, *MGMT-*promoter methylation, and *IDH* mutation. This demonstrates the collective influence of these factors on patient outcomes, with longer-surviving patients typified by multiple favorable characteristics simultaneously rather than isolated favorable factors.

### CEcore/NE volume ratio demonstrated independent prognostic value

Having identified the ratio between contrast-enhancing and non-enhancing tumor volumes as a potential biomarker, we conducted a comprehensive survival analysis to confirm its prognostic significance.

**Fig. 2:**
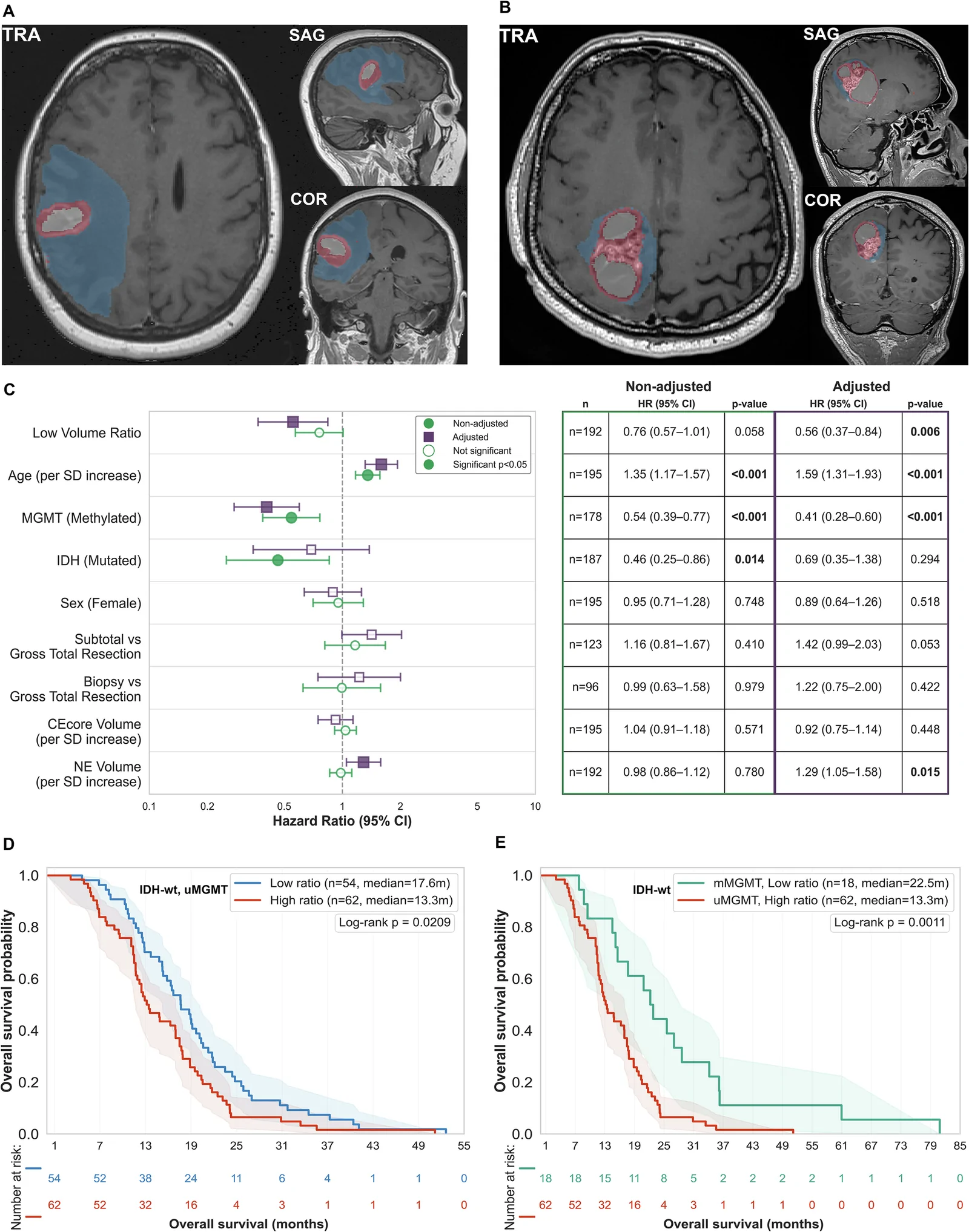
The CEcore/NE volume ratio: representative imaging examples of low- and high-ratio tumor morphology and the prognostic value for overall survival. **A** Representative example of a tumor with a low contrast-enhancing to non-enhancing (CEcore/NE) volume ratio, reflecting a non-enhancing-dominant composition associated with better overall survival. Magnetic resonance imaging (MRI) is shown in transversal (TRA), sagittal (SAG), and coronal (COR) views with pseudo-colored tumor segmentations overlaid: the non-enhancing (NE) compartment in blue, the contrast-enhancing (CE) compartment in red, and the necrotic core in white. **B** Representative example of a tumor with a high CEcore/NE volume ratio, in which the CEcore volume is similar to that in panel A but with a comparatively small NE volume. Views and color coding are as in (**A**). **C** Forest plot of Cox proportional hazards regression for overall survival, with numerical results in the accompanying table. Variables included low CEcore/NE volume ratio (versus high), age (per standard deviation [SD] increase), O⁶-methylguanine-DNA methyltransferase (MGMT) promoter methylation status, isocitrate dehydrogenase (IDH) mutation status, sex (female versus male), extent of resection (subtotal versus gross total resection; biopsy versus gross total resection), CEcore volume (per SD increase), and NE volume (per SD increase). Non-adjusted hazard ratios (HRs) are shown as green circles and adjusted HRs as purple squares, with 95% confidence intervals (CIs) indicated by horizontal whiskers. Filled markers denote statistically significant estimates (*p* < 0.05) and open markers denote non-significant estimates. The vertical gray dashed line marks the null hazard ratio of 1. The accompanying table reports per-model sample sizes, hazard ratios with 95% CIs, and *p*-values for both non-adjusted and adjusted models. **D** Kaplan–Meier estimates of overall survival stratified by the CEcore/NE volume ratio in the subgroup of patients with both IDH-wildtype and unmethylated MGMT promoter status (uMGMT) (*n* = 116): low ratio (≤0.324) in blue (*n* = 54, median overall survival 17.6 months) and high ratio (>0.324) in red (*n* = 62, median overall survival 13.3 months). Shaded bands indicate 95% confidence intervals, and the number of patients at risk over time is tabulated below the curves in matching colors. Log-rank *p* = 0.0209. **E** Kaplan–Meier estimates of overall survival in the IDH-wildtype cohort comparing a favorable biomarker combination (methylated MGMT promoter [mMGMT] and low CEcore/NE volume ratio; green; *n* = 18, median overall survival 22.5 months) with an unfavorable biomarker combination (uMGMT and high CEcore/NE volume ratio; red; *n* = 62, median overall survival 13.3 months). Shaded bands indicate 95% confidence intervals, and the number of patients at risk over time is tabulated below the curves in matching colors. Log-rank *p* = 0.0011.

Among 192 patients with infiltrative grade 4 gliomas (excluding 3 patients with purely contrast-enhancing tumors), a low CEcore/NE volume ratio (≤0.324) was significantly prognostic of improved clinical outcomes. Overall, patients with low volume ratios, Fig. 2A, demonstrated significantly longer time to progression (median 9.8 vs 7.6 months, log-rank *p* = 0.041) and overall survival (median 18.9 vs. 16.3 months, log-rank *p* = 0.036), compared to high ratio-patients, Fig. 2B. Adjusted Cox regression analysis confirmed the independent prognostic value of low CEcore/NE ratio for overall survival (adjusted HR = 0.56, 95% CI: 0.37–0.84, *p* = 0.0060), Fig. 2C. The Cox model also confirmed established factors including *MGMT*-promoter methylation (HR = 0.44, 95% CI: 0.31–0.64, *p* < 0.001), age (HR = 1.48 per SD, 95% CI: 1.23–1.79, *p* < 0.001), and gross total resection (GTR) compared to subtotal resection (STR) (HR = 0.69, 95% CI: 0.48–0.99, *p* = 0.041), while sex and *IDH* status showed no significant associations after adjustment. Details regarding established prognostic factors in our cohort are in Supplementary S2, Supplementary Fig. 1. No significant differences in age (p = 0.9524), sex (p = 0.5434), *MGMT* status (*p* = 0.6194), or *IDH* status (*p* = 0.7568) were observed between ratio groups, Supplementary S3. GTR rates were similar (51.4% vs. 44.9% for low ratio vs high ratio groups, respectively, Fisher’s exact *p* = 0.5101), though the distribution of non-GTR procedures differed, with more biopsies (24.3% vs. 10.3%) and fewer subtotal resections (24.3% vs 44.9%) in the low ratio group (chi-square *p* = 0.0107). The adjusted Cox model controls for the extent of resection.

Notably, *IDH* mutation status showed no association with CEcore/NE volume ratio distribution (Fisher’s exact test *p* = 0.7570; Cramér’s *V* = 0.020), and Cox interaction testing confirmed independence between *IDH* status and volume ratio effects (interaction HR = 0.884, *p* = 0.7220), although the cohort was skewed towards *IDH*-wildtype. Furthermore, no Cox interaction effects were observed between CEcore/NE volume ratio and *MGMT* status (*p* = 0.0890), nor age (*p* = 0.3470). This indicates that the volume ratio contributes independently to prognosis, with cumulative rather than synergistic effects alongside established factors.

Importantly, the prognostic value of the CEcore/NE volume ratio was consistent in the unfavorable *IDH*-wildtype, *MGMT*-unmethylated subgroup (*n* = 116), where patients with low ratios had significantly longer OS (median 17.6 vs. 13.3 months, log-rank *p* = 0.0209), Fig. 2D. This demonstrates that tumor composition provides prognostic information beyond established molecular markers, even in the subgroup with the poorest expected outcomes.

The absence of statistical interaction and the cumulative nature of these independent effects supported combined risk stratification using volume ratio and *MGMT*. In the *IDH*-wildtype cohort (*n* = 160 with known *MGMT* status), a four-level Cox model combining MGMT status and volume ratio group, adjusted for age, sex, and extent of resection, with the least favorable group (unmethylated MGMT, high CEcore/NE ratio, *n* = 62) as reference, confirmed a clear prognostic effect. Patients with favorable combined biomarkers (methylated *MGMT*, low ≤0.324 CEcore/NE ratio) achieved median OS of 22.5 months [IQR 15.3–32.4] compared to only 13.3 months [IQR 11.1–19.3] for the reference group, Fig. 2E, adjusted HR = 0.36 (95% CI: 0.20–0.63, *p* = 0.0004). This 9.2-month survival difference (log-rank *p* = 0.0011) was notably larger than the 5.7-month benefit of *MGMT* status alone (*p* < 0.0001).

This substantial clinical difference reflects the cumulative combination of two independent prognostic effects: *MGMT*-promoter methylation (adjusted HR = 0.43: ~6.6 months benefit) and low volume ratio (adjusted HR = 0.69: 3.0 months benefit). Consistent results were observed in the full cohort (median 26.3 vs. 14.0 months, log-rank *p* < 0.0010; adjusted HR = 0.31, 95% CI: 0.18–0.52, *p* < 0.0001), Supplementary S4, as well as for time to progression. Distribution of CEcore- and NE-volumes across groups is shown in Supplementary Fig. 2C. No differences were found across cohorts, Supplementary S5.

In sum, these findings suggest that initial tumor composition—quantified by CEcore/NE volume ratio—along with *MGMT* and age are the strongest determinants of prognosis. The effect may be driven by underlying biological factors.

### Spatial-temporal tumor recurrence is modulated by volume ratio, MGMT and age

To elucidate the biological mechanisms driving progression, we analyzed Euclidean Hausdorff95 (HD95) recurrence distances of tumor compartments between primary and recurrent tumor, identifying distinct patterns based on tumor composition, molecular markers, and patient demographics.

**Fig. 3:**
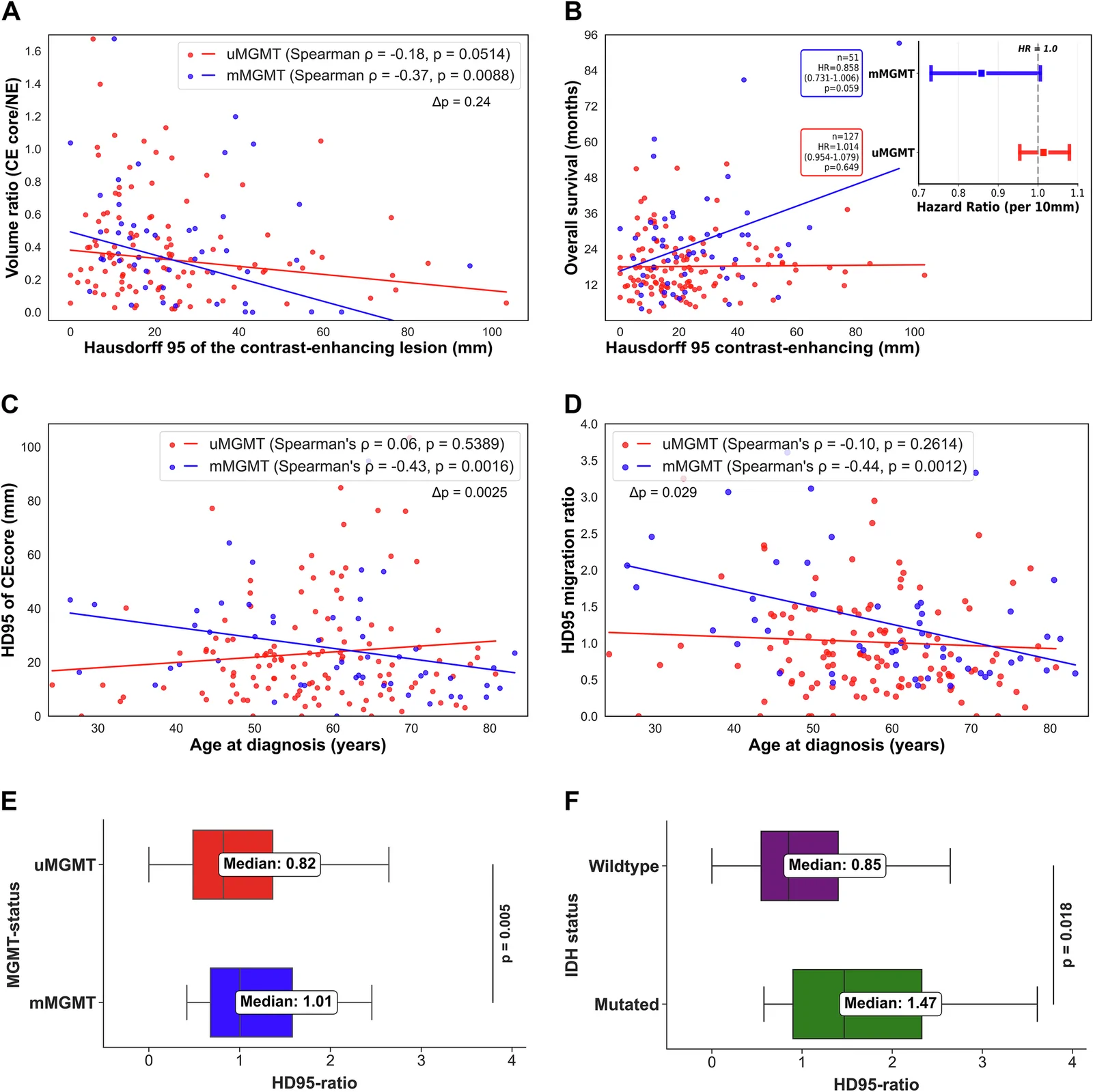
Relationships between tumor recurrence distance, primary tumor compartment composition, age at diagnosis, and overall survival, stratified by molecular status. **A** Scatter plot of the primary contrast-enhancing core to non-enhancing (CEcore/NE) volume ratio versus the 95th percentile Hausdorff distance (HD95) of the contrast-enhancing (CE) lesion, in millimeters. Patients are stratified by O⁶-methylguanine-DNA methyltransferase (MGMT) promoter methylation status: methylated MGMT (mMGMT) shown in blue and unmethylated MGMT (uMGMT) shown in red. Solid lines are linear regression fits per subgroup. Spearman rank correlations are reported in the plot legend (mMGMT: *ρ* = −0.37, *p* = 0.0088; uMGMT: *ρ* = −0.18, *p* = 0.0514). Delta *p* denotes the *p*-value from the Fisher r-to-z test for the difference in correlation coefficients between MGMT groups (*p* = 0.2400). **B** Scatter plot of overall survival (months) versus HD95 of the CE lesion (mm), stratified by MGMT methylation status (color coding as in **A**). Solid lines are linear regression fits per subgroup. The inset forest plot displays Cox proportional hazards regression hazard ratios (HRs) per 10 mm increase in HD95 with 95% confidence intervals (CIs): mMGMT (blue, *n* = 51): HR = 0.858, 95% CI 0.731–1.006, *p* = 0.0590; uMGMT (red, *n* = 127): HR = 1.014, 95% CI 0.954–1.079, *p* = 0.6490. The vertical gray dashed line marks the null hazard ratio of 1. **C** Scatter plot of HD95 of the CEcore (mm) versus age at diagnosis (years), stratified by MGMT methylation status (color coding as in **A**). Solid lines are linear regression fits per subgroup. Spearman rank correlations: mMGMT *ρ* = −0.43, *p* = 0.0016; uMGMT *ρ* = 0.06, *p* = 0.5389. Delta *p* = 0.0025. **D** Scatter plot of the HD95 migration ratio (HD95 of the CEcore divided by HD95 of the NE compartment) versus age at diagnosis (years), stratified by MGMT methylation status (color coding as in **A**). Solid lines are linear regression fits per subgroup. Spearman rank correlations: mMGMT *ρ* = −0.44, *p* = 0.0012; uMGMT *ρ* = −0.10, *p* = 0.2614. Delta *p* = 0.0290. **E** Box plot comparing the HD95 migration ratio between uMGMT tumors (red) and mMGMT tumors (blue). Median values are labeled within each box, boxes span the interquartile range, and whiskers extend to 1.5× the interquartile range. Median HD95 ratio was 0.82 in uMGMT tumors versus 1.01 in mMGMT tumors (Mann–Whitney U test, *p* = 0.0050). **F** Box plot comparing the HD95 migration ratio between isocitrate dehydrogenase wildtype (IDH-wildtype, purple) and IDH-mutated (green) tumors. Median values are labeled within each box, boxes span the interquartile range, and whiskers extend to 1.5× the interquartile range. Median HD95 ratio was 0.85 in IDH-wildtype tumors versus 1.47 in IDH-mutated tumors (Mann–Whitney U test, *p* = 0.0180).

Lower CEcore/NE volume ratios at initial diagnosis correlated with more distal recurrences of the contrast-enhancing component (Spearman’s rho = −0.23, *p* = 0.0029), with this effect most pronounced for methylated patients (rho = −0.37, *p* = 0.0088), (Fig. 3A).

For both CEcore and NE compartments, more distal recurrences correlated with longer time to progression (CEcore: Spearman’s rho = 0.157, *p* = 0.028; NE: rho = 0.174, *p* = 0.0151). These findings are notable in the context of the classical “go or grow” hypothesis^12^, and may reflect either distinct tumor-intrinsic biology or the effect of local therapy in treatment-responsive tumors. Furthermore, cox interaction analysis showed this effect predominantly was driven by *MGMT-*methylated tumors for both CEcore (*p* = 0.0488) and NE (*p* = 0.0372). Subsequent *MGMT*-stratified analysis showed that for *MGMT*-methylated tumors, CEcore recurrence distances had strong linear correlation with improved OS (Pearson’s *r* = 0.38, *R*² = 0.14, *p* = 0.0064) and clinically meaningful risk reduction (HR 0.86 per 10 mm (95% CI 0.73–1.01), *p* = 0.059), Fig. 3B. Risk reduction was consistent in NE: HR 0.82 per 10 mm (95% CI: 0.67–1.00, *p* = 0.050). Conversely, unmethylated tumors (*n* = 119) did not demonstrate survival benefit from increased recurrence distance (HR 1.02 per 10 mm, 95% CI 0.95–1.10, *p* = 0.56), suggesting distinct biological behaviors between methylated and unmethylated tumors. These findings are in alignment with previously reported survival benefit of more distal contrast enhancing recurrences among *MGMT*-methylated tumors^9,10,13^. However, we discovered that this survival benefit of increased recurrence distance in *MGMT*-methylated tumors was modulated by age with a continuous age-dependent decline (Spearman’s rho = −0.43, *p* < 0.0016), with distances decreasing by approximately 0.67 mm per year of age, Fig. 3C. This age-*MGMT* interaction was validated through adjusted analysis of covariance (ANCOVA) type II (F (1.172) = 5.55, *p* = 0.020) and permutation testing (*p* = 0.022). Segmented regression analysis identified a potential inflection point at 64.5 years of age (*F* = 2.862, *p* = 0.097). Overall, neither extent of resection nor sex showed any correlation with tumor recurrence distance, details in Supplementary S6.

There was a strong correlation between CEcore and NE recurrence distances (Spearman’s rho = 0.653, *p* < 0.001). To further investigate whether tumor compartments exhibited relational recurrence distances, we calculated the relative recurrence distance between CEcore and NE compartments, termed the HD95-ratio. An HD95-ratio >1 indicates that the contrast-enhancing component has greater spatial displacement than the non-enhancing component (CE-dominant recurrence distance), while <1 indicates NE-dominant recurrence distance. Higher HD95-ratios correlated with extended time to progression (HR 0.87 [95% CI 0.76–0.98], *p* = 0.0275). The HD95-ratio also showed age-dependent modulation, particularly in *MGMT*-methylated tumors (Spearman’s rho = −0.44, *p* = 0.001) compared to unmethylated tumors (rho = −0.10, *p* = 0.2614), Fig. 3D. This *MGMT*-age interaction effect on HD95-ratio was confirmed by adjusted ANCOVA type II (*F* (1.170) = 3.95, *p* = 0.048).

Both MGMT and IDH showed consistent effect on relative recurrence patterns. *MGMT*-methylated tumors exhibited significantly higher HD95-ratios than unmethylated (median 1.01 [IQR 0.68–1.58] vs. 0.81 [IQR 0.49–1.38], *p* = 0.006), Fig. 3E. Similarly, *IDH*-mutant tumors demonstrated higher HD95-ratios versus *IDH*-wildtype (1.47 [IQR 0.90–2.33] vs. 0.85 [0.548–1.422], *p* < 0.02), Fig. 3F. This suggest that CE-dominant recurrence patterns may reflect distinct treatment response mechanisms that ultimately affect survival.

Taken together, these findings demonstrate that when and where the tumor recurs are not random but are systematically influenced by initial tumor composition, molecular markers, and patient age.

### Anatomical recurrence patterns

**Fig. 4:**
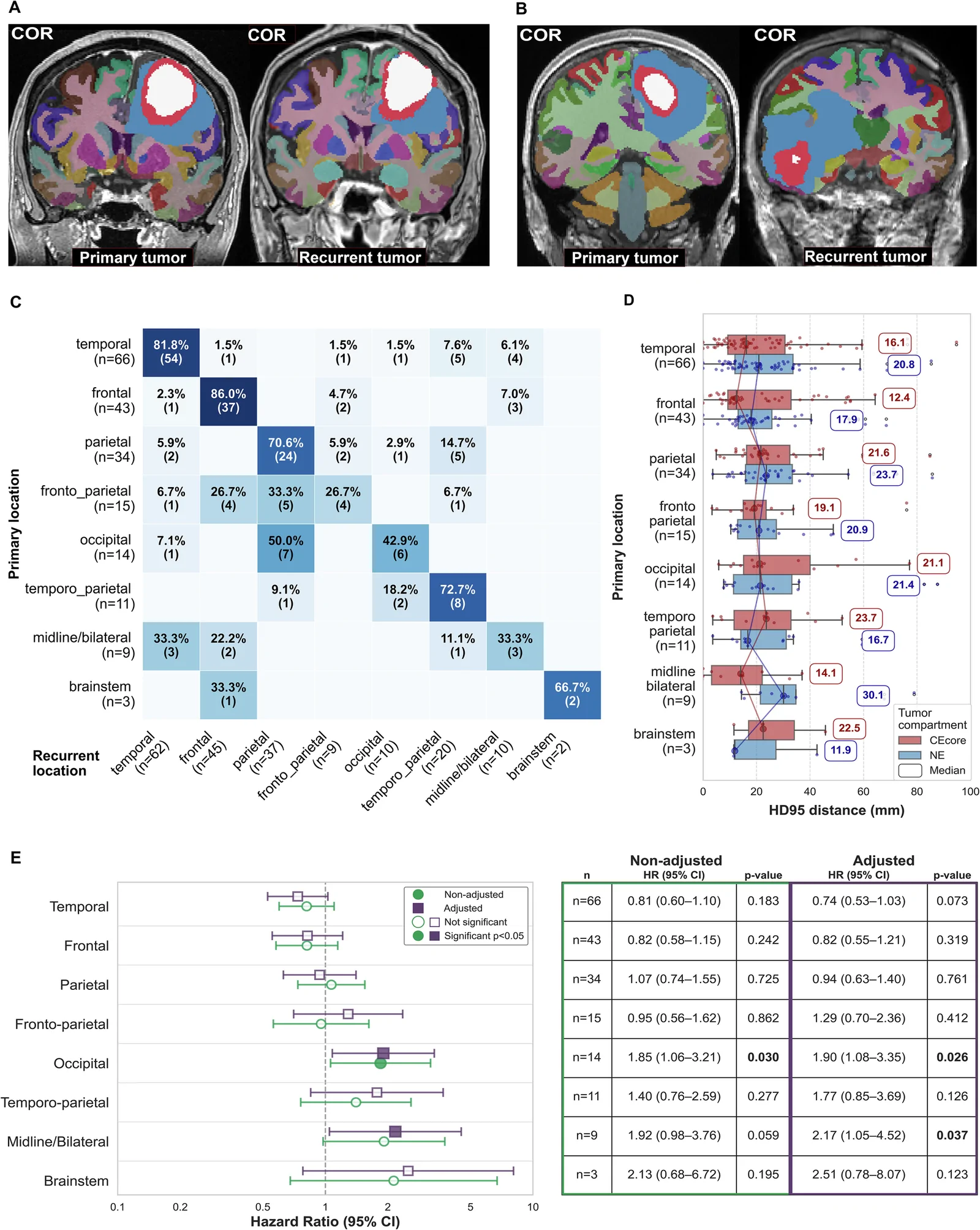
Anatomical patterns of tumor recurrence by primary lobar location. **A** Representative example of a local recurrence. Coronal (COR) post-contrast magnetic resonance imaging (MRI) of a tumor located in the frontal lobe at primary diagnosis (left) and its local recurrence in the same region (right). Tumor compartments are shown as the contrast-enhancing (CE) component in red, the non-enhancing (NE) infiltrative component in blue, and the necrotic core in white. Brain lobes are rendered with distinct color coding for cortex and white matter, derived from SynthSeg+ cortical parcellation and k-nearest neighbors (k-NN)-derived lobar white matter. **B** Representative example of a distal recurrence, shown in identical format to (**A**), illustrating a parietal tumor at primary diagnosis (left) and its cross-hemispheric temporal-lobe recurrence (right). **C** Confusion matrix of lobar tumor migration patterns from primary (rows) to recurrent (columns) locations. Cell color intensity encodes migration frequency as a percentage of the row total (darker blue indicates a higher percentage), and absolute patient counts are shown in parentheses. **D** Box plot of the 95th percentile Hausdorff distance (HD95) recurrence distances in millimeters, stratified by primary tumor location. Red boxes denote contrast-enhancing core (CEcore, including the necrotic center) distances and blue boxes denote NE distances; median values are labeled within each box, boxes span the interquartile range, whiskers indicate the observed range, and individual patient values are overlaid as points. Vertical lines connect median values across locations for each compartment. **E** Forest plot of Cox proportional hazards regression for time to progression across primary tumor locations, with numerical results in the accompanying table. Non-adjusted hazard ratios (HRs) are shown in green and adjusted HRs in purple, with 95% confidence intervals (CIs) indicated by horizontal whiskers. Filled markers denote statistically significant estimates (*p* < 0.05) and open markers denote non-significant estimates. The vertical gray dashed line marks the null hazard ratio of 1. Adjusted analyses accounted for O⁶-methylguanine-DNA methyltransferase (MGMT) promoter methylation status, isocitrate dehydrogenase (IDH) mutation status, sex, age at diagnosis, and extent of surgical resection, with each location analyzed versus all other locations combined.

To analyze progression patterns in an anatomical context, we classified tumor localization by its volumetric overlap with anatomical lobular structures at primary and recurrent timepoints. The majority of patients (*n* = 134, 68.7%) experienced local recurrence within their original anatomical lobe, Fig. 4A, while 61 (31.3%) demonstrated recurrence in new lobes in addition to locoregional recurrence, Fig. 4B. These *n* = 61 demonstrated significantly larger HD95 distances compared to local recurrences (CEcore: 26.9 mm [IQR 19.2–44.5] vs. 15.1 mm [IQR 9.9–24.1], *p* < 0.0001; NE: 26.2 mm [IQR 16.0–41.9] vs. 19.1 mm [IQR 12.1–28.3], *p* = 0.0009), confirming these anatomical transitions represented true distal recurrences.

Recurrence patterns varied significantly by primary tumor location (*χ*² = 419.05, *p* < 0.0001), with strongest migration between adjacent regions, Fig. 4C, D. Tumors originating in occipital lobe demonstrated the highest propensity for migration, with 57.1% spreading to new regions (*p* = 0.0036) and primarily to parietal areas (50.0%). Tumors originating in the parietal lobe had the second highest recurrence rate in new sites (29.4%). In contrast, frontal lobe tumors (*n* = 43) showed the most restricted recurrence pattern, with only 14.0% migrating to new regions. Temporal lobe tumors, despite being the most common initial location (*n* = 66), showed intermediate outward recurrence (18.2%), primarily to adjacent parietal regions.

These anatomical patterns had prognostic implications. Cox regression analysis revealed that primary tumor location significantly influenced time to progression, Fig. 4E. Occipital tumors carried the worst prognosis, with nearly doubled risk of progression in adjusted analyses (HR = 1.90, 95% CI: 1.08–3.35, *p* = 0.026). As expected, midline/bilateral tumors also demonstrated poor outcomes, with significantly shortened time to progression (adjusted HR = 2.17, 95% CI: 1.05–4.52, *p* = 0.037), and marked reduced overall survival (HR = 3.77, 95% CI: 3.80–7.89, *p* < 0.001). Cox regression results for *IDH*-wt cohort in Supplementary Fig. 3. HD95 recurrence distances did not differ significantly across anatomical locations after adjustment for extent of resection (ANCOVA; CEcore: *F* = 0.41, *p* = 0.8939; NE: *F* = 0.65, *p* = 0.7106), and results were consistent in fully adjusted models including MGMT, IDH, age, and sex (all *p* > 0.49). EOR itself was not associated with recurrence distance in any model, possibly due to HD95 measurements being restricted to progression outside original tumor boundary.

## Discussion

The objective of this study was to investigate the spatiotemporal recurrence patterns of grade 4 gliomas and their relationships to *MGMT, IDH*, sex, extent of resection, age at diagnosis, and survival. Understanding how tumors evolve over time and space, and how this relates to treatment response, may open new avenues for patient monitoring for early detection and intervention, as well as creating opportunities for novel combination therapies.

Two key findings emerged from our study. The primary tumor composition denoted by the CEcore/NE volume ratio, demonstrated prognostic value, with low ratios ≤0.324 independently predicting improved OS. Additive prognostic value was demonstrated when combined with *MGMT* methylation status, conferring a 9.2-month survival advantage for *IDH*-wt patients with these favorable combined biomarkers, independent of patient age. Lower ratios at primary diagnosis also correlated with more distal recurrences, suggesting that the pre-treatment tumor phenotype may influence subsequent recurrence patterns. Increased recurrence distances correlated with longer time to progression, predominantly in *MGMT*-methylated tumors, wherein the survival benefit of more distal recurrences was strongly modulated by age. Younger patients showed markedly greater benefit, and the effect diminished around 65 years, challenging the assumption that distant recurrence reflects more aggressive biology. These findings suggest that molecular-demographic factors systematically influence the spatiotemporal pattern of recurrence, with implications for individualized radiotherapy planning.

Our finding that low CEcore/NE volume ratios predict improved overall survival represents a novel quantitative imaging biomarker. In our comprehensive analysis, the CEcore/NE volume ratio emerged as one of the strongest predictors of survival outcomes, independent of established prognostic factors. Having a low CEcore/NE-ratio at diagnosis represents a favorable relationship, where the non-enhancing component is large compared to the angiogenic component. The prognostic benefit may reflect fundamental differences in tumor biology. The hyperintense T2 fluid-attenuated inversion recovery (T2-FLAIR) signal in the NE component represents a complex microenvironment containing invasive tumor cells. It is characterized by selective permeabilization of the endothelial blood-brain barrier (BBB)^14^ due to vascular endothelial growth factor (VEGF) overexpression with subsequent downregulation of claudin-5^14^ and occludin^15^ tight-junction proteins, as well as pro-inflammatory changes^16^. Increasing BBB permeability fosters vasogenic edema^14^ and, in the presence of tumor-derived chemokines, enables recruitment of immunosuppressive myeloid cells^17^. Consequently, the NE cellular milieu is enriched with tumor-associated macrophages and microglia (often 30–50% of cellularity)^18^, with interstitial fluid accumulation. Unlike the hypoxic and highly microtube-interconnected CE-compartment, NE tumor cells exist dispersed in a well-oxygenated brain parenchyma^19^ with reduced connectivity^20,21^, making them more susceptible to chemoradiation^22,23^. Although we have not found other studies investigating the prognostic value of tumor compartments’ relative sizes at diagnosis, it has been shown that NE involvement of the subventricular zone is a negative predictor of OS^24^. Furthermore, Lasocki and Gaillard noted that substantial non-enhancing tumor was more common in *IDH*-mutant grade 4 gliomas than *IDH*-wildtype glioblastoma^25^. Our work nuances this by demarcating the specific CEcore/NE threshold (≤0.324) that independently predicts improved survival, regardless of IDH mutational status. This suggests MRI tumor compartment expression may underpin subtle biological mechanisms that could inform treatment stratification.

When and where the tumor progresses has particular clinical relevance in the context of radiotherapy. Current RT protocols for grade 4 gliomas typically apply uniform margins from the contrast-enhancing lesion/surgical cavity; 15–20 mm as recommended by ESTRO-EANO^8^ and 10-20 mm as recommended by the National Comprehensive Cancer Network (NCCN)^26^, or include the whole T2-FLAIR hyperintensity volume as recommended by the Radiation Therapy Oncology Group (RTOG)^27^. These approaches are based on assumptions about infiltrative tumor behavior and ultimately, recurrence. Our HD95 measurements objectively quantify the spatial displacement between primary and recurrent tumor boundaries in 3D-space, assessing the validity of these standardized margins. We found that a longer time to progression correlated with increased recurrence distances. This observation has multiple plausible explanations. First, effective local therapy, including surgical resection and radiotherapy may achieve primary site control, so that progression manifests at distant tumor deposits rather than at the treated site. This interpretation is consistent with the previously described survival advantage associated with distant compared with local recurrence^13,28^. It also aligns with the understanding that glioblastomas diffusely involve extensive brain regions already from diagnosis. Second, the classical “go-or-grow” model posits that glioblastoma cells exist in a binary state—either highly migratory with limited proliferation, or highly proliferative with restricted migration^12^. However, Ratliff et al. recently demonstrated in vivo studies that individual glioblastoma cells can maintain both high proliferative and invasive capabilities during progression^29^. This dual capability may have multiple explanations; Kathagen and colleagues demonstrated that invasive glioma cells adapt by switching to glycolysis, making them less dependent on oxygen availability and angiogenesis^30^. This metabolic flexibility may enable cells to maintain both migratory and proliferative functions as they traverse different microenvironmental niches during progression. Heiland et al. further showed that such metabolic adaptations may be mediated by alterations in the alpha-ketoglutarate to succinate ratio, with migrating glioma cells at the tumor periphery maintaining higher alpha-ketoglutarate levels compared to cells in a hypoxic solid tumor^31^. Alternative explanations include the possibility that distal recurrences are associated with genesis of cell colonies rather than cells migrating directly from the primary tumor. Kim and colleagues demonstrated that distant recurrences share only 25% of mutations with primary tumors, indicating early divergent evolution rather than direct migration^32^. Nevertheless, our findings fundamentally challenge the “one-size-fits-all” approach to radiation margin design. Rather than viewing distant recurrence as treatment failure, it may paradoxically indicate beneficial tumor biology with local disease control and treatment response, in light of the prolonged time to progression. The higher recurrence distances we observed in *MGMT*-promoter methylated tumors align with Minniti and colleagues’ analysis showing distant recurrence in 18.6% of methylated versus 6% of unmethylated tumors^9^, and Shidoh and colleagues’ observation that progression-free survival benefit correlates with more distal recurrence in *MGMT*-methylated cases^10^. However, our strong negative correlation between age and recurrence distances (Spearman’s rho = −0.43, *p* < 0.001) in *MGMT*-methylated tumors suggest that the previously reported survival advantage of distant recurrences in *MGMT*-methylated patients may be primarily driven by younger patients, with the effect diminishing around 65 years of age. This aligns with established age-related tumor biology, including younger patients having enriched proneural molecular subtype^33^. These findings could indicate that age modulates the tumor cell’s capability to maintain a dual migrate-and-proliferate state, particularly in *MGMT*-methylated tumors. The finding also supports Hegi and colleagues’ recent demonstration that elderly patients with unmethylated *MGMT* promoter gain no benefit from TMZ treatment, concluding that molecular markers should be interpreted within the context of age for optimal treatment stratification^34^.

In sum, these results inform the ongoing debate regarding personalization of clinical target volumes (CTV) in radiotherapy planning. Several studies have found it safe to reduce RT margins to minimize neurotoxicity while maintaining tumor control^9,35–37^. However, previous studies have typically classified recurrences into categorical patterns (local, marginal, or distant) based on fixed CTV-margins or isodose expansions without age-stratified analysis of spatial recurrence patterns. Our study quantifies recurrence distances in millimeters as a continuous variable, enabling granular assessment of tumor recurrence dynamics in the context of molecular and demographic covariates. Our findings suggest that the safety and efficacy of margin reduction may depend critically on the interplay of these factors, and support two distinct clinical scenarios that warrant prospective evaluation. First, younger patients harboring *MGMT*-methylated tumors with low volume ratio demonstrate increased recurrence distances, prolonged time to progression, and improved overall survival—a pattern consistent with enhanced treatment response. For these patients, a clinical dilemma arises: large CTV-margins may seem inappropriate for long-survivors in light of the potential RT-induced risk of cognitive impairment that develops over time. On the other side, these patients may have achieved their survival benefit from standard large margins that capture dispersed tumor cells that remain treatment-sensitive in the well-vascularized brain parenchyma. Whether margins can be safely modified in this subgroup—potentially through adaptive strategies informed by longitudinal imaging—requires investigation in a prospective setting. Second, older patients and those with unmethylated *MGMT*-promoter and high CEcore/NE volume ratio tumors demonstrate rapid, localized progression, indicating limited benefit from wide margins. These patients may be candidates for more focused radiation with reduced margins, which could allow steeper dose gradients to optimize local control while minimizing irradiated volume. This approach may be particularly relevant given that elderly or low-KPS patients often receive reduced doses due to increased toxicity risk.

The strong interaction between CEcore/NE ratio, *MGMT* status, *IDH* status, and age demonstrates that radiation planning should incorporate multiparametric risk assessment rather than uniform approaches. Importantly, future margin-reduction studies should account for these combined effects.

Additionally, the distinct recurrence patterns in *MGMT*-methylated tumors suggest therapeutic strategies modulating MGMT levels, including *MGMT* promoter silencing through epigenetic modifiers or proteasome inhibitors like bortezomib^38^ may potentially impact personalized selection of RT margins.

The distinct recurrence patterns we found across lobular locations could reflect underlying anatomical landscape that guides tumor cell invasion and may support anatomically-informed radiation planning strategies. Occipital tumors’ high migration probability (57.1%) may be facilitated by the rich interconnectivity of posterior white matter, including the splenium of the corpus callosum and posterior commissural pathways, supporting previous observations that posterior tumors possess distinct biological behavior^39,40^. Occipital tumors’ propensity for migration combined with shortened time to progression (HR = 1.90), suggests these patients may benefit from wide initial margins. Conversely, the more restricted migration of frontal (14.0%) and temporal tumors (18.2%) could reflect the more confined anterior white matter architecture, consistent with Smith et al., who demonstrated that temporal lobe tumors typically follow white matter tracts within the lobe rather than crossing to adjacent regions^41^.

However, cerebral lobes differ substantially in volume, whereas tumors originating in smaller lobes are geometrically more likely to extend beyond lobular boundaries, which may partly account for the high migration rate observed in occipital tumors. Additionally, although our ANCOVA analysis demonstrated that EOR did not significantly predict HD95 distance, and Cox regression for TTP by location was adjusted for EOR (Fig. 4E), the study may be underpowered to fully capture such effects.

The primary strengths of this study include its large, multi-center cohort with paired primary and recurrent MRIs, the substantial, well-annotated quantitative imaging data, and the comprehensive longitudinal clinical data from the Norwegian health records with limited loss to follow-up.

Limitations include the retrospective design with inherent selection bias toward patients with repeated imaging. Formal Response Assessment in Neuro-Oncology (RANO) 2.0 quantitative thresholds were not applied for recurrence diagnostics, as the retrospective, multi-center design predates the routine implementation of these criteria. The observed GTR rate of 48% (71/148 with known EOR status) is consistent with population-based Norwegian data reporting GTR rates of 36–48%, reflecting the unselected nature of our cohort compared to high-volume referral series^42,43^. Missing clinical data may have influenced specific subgroup analyses. Additionally, inherent methodological challenges in longitudinal image analysis should be acknowledged. Despite state-of-the-art registration techniques, no co-registration method can fully account for all morphological changes occurring between primary diagnosis and recurrence, such as surgical cavity evolution, brain shift, and treatment-induced tissue alterations. This introduces some uncertainty in HD95 measurements. However, the magnitude of observed effects and consistency across multiple analyses suggests robust underlying patterns despite these technical limitations. The study also lacks integration with whole molecular profile, and proven causal mechanisms through prospective validation. The CEcore/NE volume ratio should be validated on an independent cohort.

An ongoing prospective study including whole exome sequencing is investigating longitudinal MRI-expressions, including the CEcore/NE volume ratio at diagnosis, with machine learning prediction models, aiming to validate our findings with objective endpoints. Future research should focus on integrating comprehensive molecular profiling with spatiotemporal imaging features to develop more refined predictive models for tumor progression and treatment response.

In conclusion, this multi-center study comprising 195 patients and 390 paired MRI examinations has demonstrated three findings that may offer new directions for personalized treatment. First, that the volume ratio of the initial tumor’s CEcore- relative to NE-volume is independently prognostic, with ratios ≤0.324 predicting significantly improved overall survival. Second, tumor spatial recurrence patterns in *MGMT-*promoter methylated tumors from younger patients exhibited more distal recurrences. Third, increased recurrence distances in both CEcore and NE tumor compartments correlated with prolonged survival, specifically in *MGMT*-promoter methylated patients, suggesting that spatial progression patterns reflect underlying treatment responsiveness. These findings provide a foundation for molecular-demographic risk stratification and precision radiotherapy planning in grade 4 gliomas.

## Methods

### Study design and participants

We analyzed paired multiparametric MRI (mpMRI) datasets from 195 patients with WHO grade 4 gliomas^44^, in total comprising 390 MRI examinations from primary diagnosis and first recurrence for each patient. The study population comprised *n* = 57 subjects who had given written consent by January 2025 in our ongoing phase I/II clinical trial (ClinicalTrials.gov Identifier: NCT03643549) from three regional health authorities in Norway: Haukeland University Hospital (HUH), Oslo University Hospital (OUH) and St Olavs (Trondheim University Hospital, TUH). Additionally, we included *n* = 97 age–matched control subjects from HUH (January 2015–January 2025), as well as 41 subjects with repeated imaging from The Cancer Imaging Archive (TCIA) glioblastoma open access dataset, provided by the University of Pennsylvania (UPENN)^45^. This Research involving human research participants, their biological material, and medical data was performed in accordance with the Declaration of Helsinki, Good Clinical Practice requirements and all work involving databases of human information, electronic or otherwise, including genetic information adhered to the European directive 95/46 CE and the data protection national law enforced by Norway (http://www.datatilsynet.no). Ethical approval was obtained from the Regional Ethics Committee in Western Norway (reference number 2017/2084/REK vest), with participating centers in South-Eastern and Central Norway operating within this approval, in addition to approval from the Norwegian Medicines Agency (17/17445-17). Written informed consent was obtained by signature from all NCT03643549 trial participants. For the contemporary control group, the requirement for informed consent was waived by the Regional Ethics Committee in Western Norway, in accordance with the Norwegian Health Research Act §35, as the study involved retrospective analysis of previously treated, deceased patients, where obtaining consent was not feasible and the research was considered to entail minimal risk with no impact on patient welfare or rights. For the TCIA cohort, the ethical approval was issued by the local review board at UPENN.

This study addressed the NCT03643549 trial’s secondary objective regarding imaging biomarkers using retrospective MRI data acquired from primary diagnosis and first recurrence - both timepoints occurring prior to trial enrollment and administration of bortezomib + temozolomide sequential combination therapy.

**Fig. 5:**
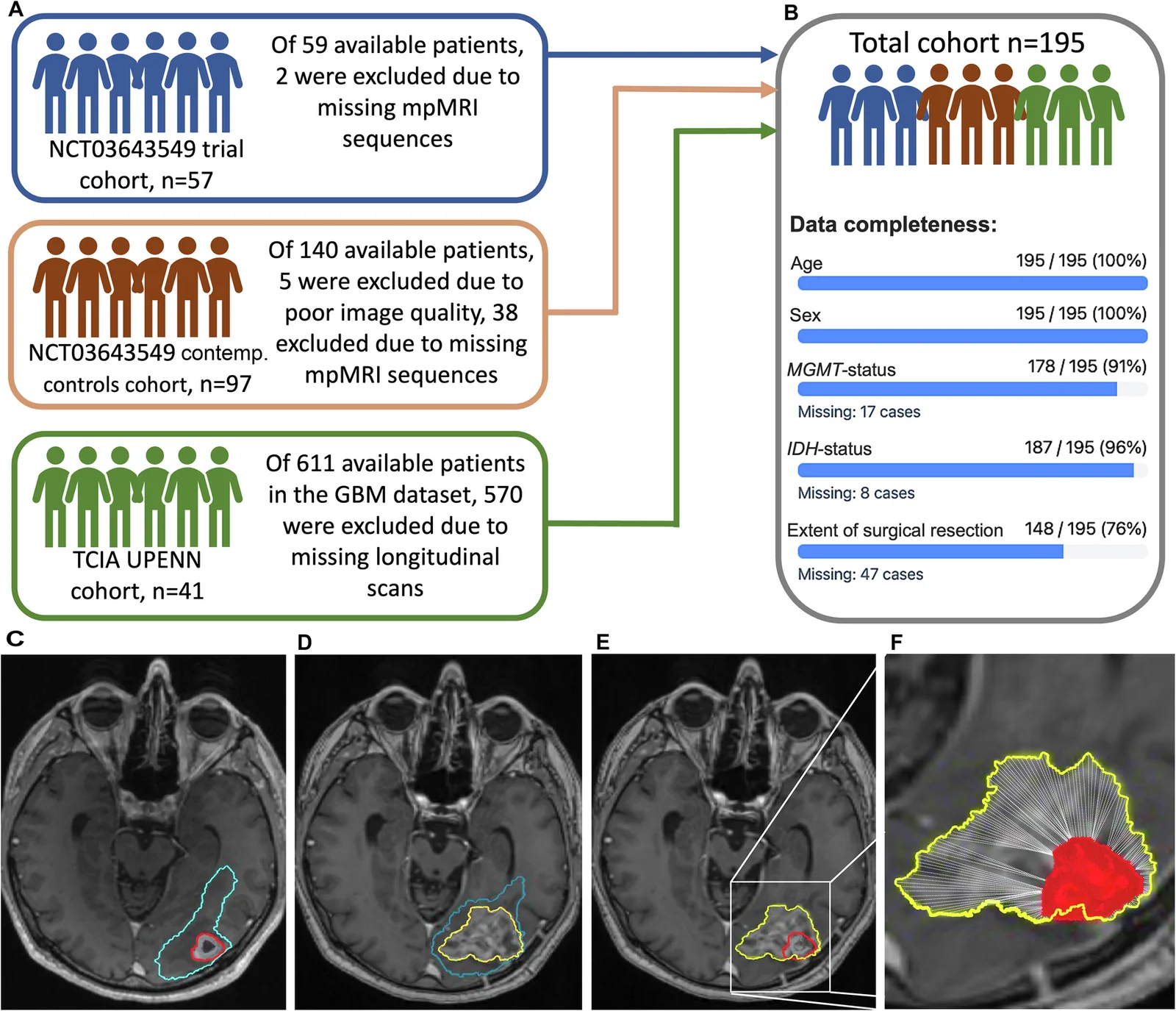
Overview of the study cohorts, data completeness, and the spatiotemporal recurrence distance mapping workflow. **A** Schematic of the three contributing study cohorts, showing the number of available patients, the number of and reasons for exclusions, and the final sample size from each cohort: the NCT03643549 trial cohort (*n* = 57; 2 of 59 available patients excluded due to missing multiparametric magnetic resonance imaging [mpMRI] sequences), the NCT03643549 contemporary controls cohort (*n* = 97; of 140 available patients, 5 excluded due to poor image quality and 38 excluded due to missing mpMRI sequences), and The Cancer Imaging Archive (TCIA) University of Pennsylvania (UPENN) cohort (*n* = 41; of 611 available patients in the glioblastoma [GBM] dataset, 570 excluded due to missing longitudinal scans). Colored arrows link each cohort to the pooled study population in (**B**). **B** Summary of the total study cohort (*n* = 195) with horizontal bar charts indicating data completeness across demographic variables (age, sex), molecular markers (O⁶-methylguanine-DNA methyltransferase [MGMT] promoter methylation status, isocitrate dehydrogenase [IDH] mutation status), and extent of surgical resection. The number of cases with available data, the corresponding percentage, and the number of missing cases are reported for each variable. **C** Axial post-contrast T1-weighted MRI at primary diagnosis showing tumor segmentation: the contrast-enhancing (CE) tumor component boundary is outlined in red, and the non-enhancing (NE) tumor component boundary is outlined in cyan. **D** Axial slice-matched MRI at established recurrence, with the recurrent CE tumor compartment boundary outlined in yellow and the recurrent NE tumor compartment boundary outlined in cyan. **E** Overlay of the primary CE compartment boundary (red) onto the recurrent CE compartment boundary (yellow), visualizing the spatial relationship between primary and recurrent disease across time. The white rectangle indicates the region magnified in (**F**). **F** Magnified conceptual illustration of the Euclidean distance transform map. White lines indicate, for each voxel on the recurrent tumor boundary (yellow), the shortest distance to the nearest voxel on the primary tumor boundary (red), measured across the three spatial dimensions (x, y, z).

Inclusion criteria were: (i) age ≥18 years, (ii) oncologist confirmed recurrence or progression by longitudinal scans to rule out pseudoprogression, (iii) available diagnostic-quality mpMRI from both initial diagnosis and first recurrence, (iv) histologically and radiologically confirmed primary and recurrent WHO grade 4 glioma, (v) received standard Stupp protocol^8,46^ at their primary diagnosis, (Fig. 5A, B).

Our cohort includes both glioblastoma, *IDH*-wildtype (*n* = 176) and astrocytoma, *IDH*-mutant, grade 4 (*n* = 11), with 8 cases of inconclusive *IDH* status. Both entities are classified as WHO grade 4 diffuse gliomas and received identical initial treatment (Stupp protocol). *IDH* mutation status is included as a covariate in all adjusted analyses, and all primary findings were replicated in the *IDH*-wildtype subgroup.

### Outcomes

The primary outcome was HD95 recurrence distances in relation to *MGMT*-promoter methylation and *IDH* mutation status, age, sex, EOR, and the CEcore/NE volume ratio composition of the primary tumor. Secondary outcomes were (i) anatomical recurrence patterns between brain lobes from primary tumor to recurrent disease, and (ii) and identification of features predicting survival.

### Data collection

Clinical data were sourced from the Norwegian electronic health record system ‘DIPS’. For the NCT03643549 cohort and historical controls, mpMRI data were automatically anonymized using the built-in de-identification tool and exported in DICOM format from the Picture Archiving and Communication Systems (PACS) at each study site. The anonymized DICOM files were converted to NIfTI format for further analysis^47^. MRI- and clinical data were stored anonymously on the Secure Access to Research Data and E-Infrastructure (SAFE) facility at the University of Bergen.

For each patient, we collected sex, age at diagnosis, *IDH* mutation and *MGMT*-promoter methylation status, EOR, as well as time to progression (TTP) and overall survival (OS), Fig. 5B. EOR were categorized as gross total resection (defined from surgical records as removal of all visible contrast-enhancing tissue), subtotal resection (categorized as any residual tumor), or biopsy. Surgical records were missing for *n* = 41 UPENN cases where EOR is not known, and inconclusive GTR/STR status on *n* = 6 Norwegian patients, data collection pre-dating RANO-RESECT criteria^48^. mpMRI included T1-weighted pre- (T1) and post-contrast (T1c), T2-weighted (T2) and T2-FLAIR.

Recurrence was established by radiologically confirmed progressive tumor growth after completion of standard primary treatment. This was determined by radiologist and oncologist assessment based on serial imaging follow-up, and histopathology reports where available. Pseudo-progression was ruled out by subsequent scans confirming active progression, providing diagnostic certainty.

### Image analysis processing

The image-analysis pipeline comprised five sequential steps detailed below, briefly: (i) intra-patient affine registration of all mpMRI sequences to the post-contrast T1-weighted reference, (ii) deep learning tumor segmentation, (iii) inter-timepoint diffeomorphic registration of paired primary and recurrence datasets, (iv) anatomical brain parcellation with subsequent anatomical tumor localization classification, and (v) quantification of spatial recurrence distance using the 95th percentile Hausdorff distance between primary and recurrent tumor boundaries.

#### Intra-dataset multiparametric MRI registration

All n = 390 mpMRI datasets underwent re-slicing and affine registration to each dataset’s post-contrast T1-weighted sequence using EasyReg^49^ to align with each patient’s post-contrast T1-weighted sequence coordinate system and voxel dimensions. EasyReg is a modality-agnostic and resolution-agnostic registration model that implements a diffeomorphic framework with stationary velocity fields, which preserves topology and ensures inverse consistency^49^. Prior to computing deformation fields, input images were resampled to a standardized 1 mm isotropic grid in the Montreal Neurological Institute (MNI) reference space, automatically accommodating variations in slice thickness across different image weightings. This registration process ensured precise spatial alignment of voxels across the multiparametric MRI acquisitions, establishing a consistent in-patient geometry for subsequent image analysis while compensating for patient movement during MRI acquisition. The post-contrast T1-weighted sequence served as the reference image due to its consistently superior resolution and image quality compared to other sequences in the imaging protocol.

#### Deep learning tumor segmentation

Tumor segmentation was performed using the pre-trained “segment_glioma” model^50^ based on deep learning nnU-Net (“no-new U-Net”) framework^51^, providing three tumor compartment predictions: (i) the necrotic core, (ii) the contrast enhancement (CE) of actively growing tumor cells in areas of disrupted blood-brain-barrier (BBB), and (iii) the non-enhancing (NE) T2-FLAIR hyperintense component consisting of tumor cells along with vasogenic edema from vascular permeability alterations and inflammatory responses. The model selection was based on previous robust performance in peer-reviewed literature^50–52^ and internal validation that confirmed its robustness in producing high-quality segmentations with maintained consistency across diverse image matrices and common clinical imaging artifacts - including motion artifacts, aliasing, and Gibbs ringing. The segment_glioma model was trained on the PICTURE dataset (*n* ≈ 500) collected from 12 European hospitals, representing an architectural advancement from its predecessor, HD-GLIO, which was trained on a larger corpus of *n* ≈ 3200 datasets^53^. This deep learning framework employs a multi-modal approach by simultaneously training three distinct U-Net variants (2D, 3D, and cascaded models), and employs cross-validation to automatically select the optimal configuration for each specific case.

The model’s architecture is based on the U-Net design, featuring a convolutional neural network with an encoder-decoder structure enhanced by skip connections. To balance spatial context with computational efficiency, it processes volumetric MRI data in patches (typically 128×128×128 voxels). The framework incorporates complementary analysis strategies: the 2D configuration independently analyzes axial slices, while the cascade model employs a multi-resolution approach, first capturing global context at lower resolution before refining predictions at full resolution. We selected segment_glioma as the direct successor to the HD-GLIO model, which our team has previously validated through both retrospective and prospective testing, demonstrating its clinical feasibility in our hospital setting^54^.

All segments were visually inspected by a professional medical dosimetrist with 20 years’ experience. The combined CE and tumor core volumes were assessed as ‘CEcore’ for further analysis.

#### Inter-timepoint deformable co-registration

Registration between each patient’s paired MRI datasets from initial diagnosis and tumor recurrence was performed using ‘Advanced Neuroimaging Tools’^55^ (ANTs). We applied the ANTs framework with symmetric normalization (SyN) transformation, which generated diffeomorphic deformation fields while preserving inverse consistency between the longitudinal timepoints. This approach ensured anatomical correspondence despite structural changes over time. Nevertheless, post-surgical brain shift and tissue loss represent inherent challenges, requiring manual assessment and corrections/iterations case by case. Our manual control of each registration assessed the alignment and coherence of anatomical landmarks, Supplementary Fig. 4. For co-registering the tumor segmentation maps, we employed nearest neighbor interpolation during transformation between timepoints to maintain the discrete classification boundaries of tumor regions. This interpolation method was critical for preserving segmentation integrity and preventing partial volume effects that could otherwise blur the boundaries between distinct tumor compartments and compromise volumetric and spatial analyses. The absolute Euclidean distances between primary and recurrent tumor boundaries were measured using the 95th percentile Hausdorff distance (HD95) for CEcore and NE. A 3D distance transform was computed for the recurrent tumor mask, creating a point-to-surface distances map for each *recurrent* voxel’s shortest distance to the nearest *primary* tumor boundary, Fig. 5C–F. This approach inherently accounts for complex 3D tumor geometries while excluding outliers. Recurrent tumor boundary not exceeding outer primary tumor boundary was designated zero mm. This methodological decision was made to suppress the effect from intra-cavity regrowth, i.e., after incomplete surgical resection.

#### Anatomical brain parcellation and tumor localization

The post-contrast T1-weighted (T1c) images underwent processing through SynthSR to generate high-resolution T1-weighted reconstructions^56^. We specifically selected T1c imaging for this purpose due to its superior gadolinium-enhanced signal-to-noise ratio and excellent soft-tissue contrast properties, while effectively minimizing potential confounding effects from enhancing tumor regions. This crucial pre-processing step established the foundation for subsequent comprehensive 3D morphometric analysis using the robust SynthSeg+ model, which is built on an advanced 3D U-Net architecture^57,58^. The SynthSeg+ implementation generated a detailed anatomical parcellation comprising 101 distinct anatomical labels: 68 cortical parcellations distributed across left and right hemispheres (e.g., “ctx-lh-inferiorparietal”, “ctx-rh-caudalanterior-cingulate”), 28 subcortical structures (e.g., hippocampus, thalamus), and 5 central structures (e.g., brainstem). This comprehensive segmentation provided a detailed anatomical framework for tumor localization analysis. However, we encountered a significant limitation for our tumor-overlap analysis: SynthSeg+ only generates white matter (WM) segmentations as unified left or right hemispheric volumes without more granular lobular differentiation. To address this limitation and enable a more precise anatomical localization, we implemented a refined post-processing approach. Using a K-nearest neighbors’ algorithm (*K* = 3), we further classified each hemispheric WM voxel into specific lobular regions based on its spatial proximity to corresponding groups of cortical parcellations. This additional processing step enabled a comprehensive assessment of tumor overlap with specific anatomical brain regions, Supplementary Fig. 5.

Classification of anatomical tumor localization was performed by analyzing overlapping CEcore- and anatomical lobe voxels, considering frontal, parietal, temporal, and occipital lobes, as well as brainstem/cerebellum regions and midline/bilateral patterns. To establish optimal classification thresholds, we implemented a multi-metric optimization approach that balanced four complementary evaluation metrics: silhouette score (quantifying cluster separation and cohesion), entropy score (measuring distribution balance across categories), mean confidence (assessing average classification certainty), and high-confidence rate (calculating the proportion of high-certainty classifications). These metrics were integrated into a weighted composite score with manually calibrated contributions (30% silhouette, 20% entropy, 30% mean confidence, 20% high-confidence rate). We determined optimal thresholds through comprehensive grid search across parameter ranges (junction thresholds: 0.05–0.35; dominant lobe thresholds: 0.80–1.0). This systematic evaluation yielded optimal threshold values of 0.9 for dominant lobe classification and 0.24 for junction classification. Based on these thresholds, we classified tumors as “single-lobe dominant” when ≥90% of CEcore voxels overlapped with a single anatomical lobe. Conversely, tumors were classified as “bilateral” or “junction” when >24% of CEcore voxels overlapped with both hemispheres or adjacent lobes, respectively. Borderline cases were assigned to the nearest classification category based on proximity to the established threshold. Classification achieved median confidence scores of 0.92 and 0.95 for primary and recurrent tumors, respectively, and formed the basis for anatomical tumor trajectories analysis across timepoints.

### Statistical analysis and software

All analyses were performed on the full *n* = 195, unless stated otherwise. Minimum sample size was determined by power calculation. To detect an expected mean difference in recurrence distance of 10 mm (SD = 20 mm) with *α* = 0.05, *n* = 143 provides 80% power. We therefore aimed for minimum *n* = 143, while including all eligible patients to retain sufficient power for potentially smaller effects. Missing data were handled by complete case analysis for respective analyses, Fig. 5B, while cases were retained for other analyses. Survival probabilities were calculated using Kaplan-Meier analysis^59^ with log-rank tests for multiple group comparisons, while risk factors were analyzed using non-adjusted and adjusted Cox proportional hazards regression models^60^. Adjusted Cox regression analysis was performed on *n* = 168 with complete *MGMT, IDH*, age, CEcore/NE volume ratio and EOR. Resection categories included gross total resection, subtotal resection, biopsy^61^, as well as ‘unknown’ to avoid power loss. As all patients were deceased at time of analysis, there were no censored observations in survival analyses. Correlations were assessed using Pearson’s- or Spearman’s rank correlation coefficient, and group comparisons used log-rank or Mann-Whitney U tests for continuous variables and chi-square or Fisher’s exact tests for categorical variables. Corrections for multiple comparison used Benjamini-Hochberg method^62^. Adjusted comparison of mean values was performed using ANCOVA, type II. Data processing and analyses were performed using Python version 3.11.9 (Python Software Foundation, Wilmington, Delaware, USA). Survival analyses were performed in Stata version 18 (StataCorp LLC, College Station, Texas, USA).

### CEcore/NE threshold derivation

Volumetric analysis assessed tumor segmentations as dual tumor compartments; CEcore and NE. We evaluated their proportionate relationship through the CEcore/NE volume ratio, analyzed both as continuous and binary variables. The optimal binary threshold of 0.324 was identified using maximally selected rank statistics, and rigorously validated through complementary approaches: (i) bootstrap validation using 100 resampled datasets confirmed threshold reproducibility (mean 0.335, 95% CI: 0.200–0.440), (ii) quartile cross-validation analysis across different percentile splits (25th, 33rd, 50th, 67th, 75th) demonstrating consistent optimal thresholds within the 25–33rd percentile range, and (iii) information criteria comparison strongly favoring the binary model with lowest Akaike Information Criterion (AIC) and Bayesian Information Criterion (BIC). To validate our analytical approach, we performed comprehensive restricted cubic splines analysis testing 3–8 knots positioned at quantile-based locations, Supplementary Fig. 6. Model selection employed multiple criteria, including C-index for discriminative ability, AIC for penalized likelihood, and BIC for conservative model selection. These comprehensive analyses confirmed threshold-based rather than continuous relationships with survival outcomes, supporting a binary classification approach. Given the absence of linear relationships, we proceeded with threshold-based analysis. Importantly, to verify that the prognostic effect was specifically attributable to the volume ratio rather than driven by its individual components, we conducted separate analyses for CEcore and NE volumes independently. Firstly, we conducted a log-rank test to see if having tumor component size below or over median associated with significantly difference in survival (log-rank *p* = 0.61for CEcore, *p* = 0.24 for NE). Secondly, we included the individual volumes in Cox regression analysis to transparently report the effect of individual tumor compartment volumes in unadjusted and adjusted analysis.

### Machine learning analysis

To systematically identify predictive factors and their relative contributions to tumor behavior, we implemented a comprehensive machine learning framework. Using the PyCaret machine learning pipeline, we evaluated 19 distinct regression models through rigorous 90–10 train-test splitting and 5-fold cross-validation protocols. The Huber Regressor consistently emerged as the optimal model across all prediction targets, likely due to its robustness against outliers in our heterogeneous clinical dataset. In PyCaret, the Huber Regressor is a robust regression model that is less sensitive to outliers than ordinary least squares regression. It is based on Huber loss, which combines the advantages of squared loss (for small errors) and absolute loss (for large errors), making it particularly useful for datasets with heteroscedasticity or outliers.

Our predictive models incorporated a multidimensional feature set including established molecular markers (MGMT methylation status, IDH mutation status), demographic factors (age), clinical parameters (EOR), imaging characteristics (initial tumor volumes of contrast-enhancing and non-enhancing components and their ratio), and anatomical tumor location. This integrated approach enabled us to evaluate the relative contribution of each feature to various clinical outcomes. Machine learning analysis served primarily for pattern detection and exploratory feature ranking, before examining specific relationships with conventional statistical methods.

## Supplementary information

Supplementary Information

## Data Availability

The data from the cohorts NCT03643549 trial (*n* = 57) and historical controls (*n* = 97) generated in this study are sensitive, thus not publicly available due to patient privacy protection requirements and ethical restrictions imposed by the Regional Ethics Committee in Western Norway (reference number 2017/2084/REK vest). Data are available upon reasonable request from the corresponding author MC for researchers who meet the criteria for access to confidential data. The data from the TCIA UPENN cohort (*n* = 41) are openly available from https://www.cancerimagingarchive.net/collection/upenn-gbm/.

## Code availability

The code for the complete image analysis, deep learning tumor segmentation, anatomical segmentations and classification as well as machine learning, is publicly available on GitHub: https://github.com/MarianneHannisdal/spatiotemporal-glioma.
