# Supplementary material for: Spatial-temporal recurrence patterns of grade 4 glioma using deep learning integrated multiparametric MRI and molecular pathology
Source: NPJ Precis Oncol. 2026 Jun 13;10:364. doi: 10.1038/s41698-026-01557-0 (PMC13620126; doi:10.1038/s41698-026-01557-0)
Supplement: Supplementary file 1 [file 41698_2026_1557_MOESM1_ESM.pdf]

Spatial temporal recurrence patterns of grade 4 glioma using deep learning integrated multiparametric MRI and molecular pathology

Marianne H Hannisdal, Arvid Lundervold, Dorota Goplen, Jorunn Brekke, Mohummad A Rahman, Petter Brandal, Tora Solheim, Stein Atle Lie, Nello Blaser, and Martha Chekenya

Supplementary appendix

S1: Machine learning performance metrics 2
S2: Established prognostic factors in our cohort 4
S3: Group sensitivity analysis 7
S4: CEcore/NE volume ratio: full cohort vs *IDH* wildtype cohort 8
S5: Cohort sensitivity analysis 9
S6: HD95 in relation to EOR and anatomical location and sex 11
S7: Cox regression of overall survival by primary tumor location 11
S8: Co-registration across time-points 12
S9: Anatomical tumor trajectory 13
S10: Derivation of the CEcore/NE ratio threshold 14

S1: Machine learning performance metrics

Performance metrics is summarized in supplementary table 3:

Supplementary Table 1:

PREDICTION PERFORMANCE METRICS - EVALUATION ON TEST SET						
	MAE	MSE	RMSE	R2	RMSLE	MAPE
HD95	14.2915	414.2499	19.4079	-0.0914	0.8911	0.8875
TIME TO PROGRESSION	5.1126	62.5752	7.7237	-0.0767	0.5652	0.6399
OVERALL SURVIVAL	8.4070	193.8195	13.1395	-0.0940	0.5328	0.4998

Supplementary Table 1: Machine learning model performance metrics for predicting tumour migration and survival outcomes in grade 4 glioma. *Performance metrics for Huber Regressor models trained to predict three outcomes - 95th percentile Hausdorff distance (HD95) between primary and recurrent contrast-enhancing tumour cores, time to progression, and overall survival - evaluated on the held-out test set (n=175 training, n=20 testing). Reported metrics are Mean Absolute Error (MAE), Mean Squared Error (MSE), Root Mean Squared Error (RMSE), coefficient of determination (R^2), Root Mean Squared Logarithmic Error (RMSLE), and Mean Absolute Percentage Error (MAPE).*

Discussion: Machine learning metrics

The interpretation of model performance metrics warrants careful consideration due to several factors specific to this clinical dataset, and to the purpose of the machine learning analysis. First, it's important to note that all reported metrics are based on a relatively small test set (n=20 patients), representing 10% of our total cohort. While this split ratio is methodologically sound for the exploratory purpose (pattern detection and exploratory feature ranking and not as predictive models), the limited test set size means performance metrics should be interpreted with appropriate caution. We wish to emphasize that the manuscript's conclusions are not, and should not be, based on the predictive power of the ML model. Our workflow using ML to perform exploratory feature ranking, followed by traditional statistical validation is to our knowledge a standard approach in modern bioinformatics and biomarker discovery. All clinically relevant findings and conclusions are based on our subsequent validated statistical analyses (Cox regression, correlation analyses, log-rank tests) rather than the machine learning models themselves.

Furthermore, the nature of our outcome variables presents inherent predictive challenges. Survival outcomes in grade 4 glioma patients exhibit exceptionally broad ranges - from as short as a few months to several years -

creating a distribution with substantial variability. This heterogeneity, combined with the complex, non-linear relationships between clinical features and outcomes, makes prediction against median values particularly difficult. The poor R^2 values, as detailed in Supplementary Table 1, likely reflect the inherent complexity and heterogeneity of glioma biology rather than methodological inadequacy. The consistent negative R^2 values suggest complex non-linear relationships that require larger cohorts for robust prediction.

Despite these limitations in absolute predictive accuracy, the validity of the ML component is nevertheless strengthened by its ability to identify key features. The models successfully identified primary tumor composition, molecular markers, and age as strong determinants of both migration patterns and survival outcomes—findings we subsequently validated through targeted statistical analyses. The consistent identification of key features across multiple models provides valuable insights into the relative importance of specific predictors. These insights remain valuable even when the model's overall predictive power is modest, pointing to biologically meaningful relationships that warrant further investigation in larger cohorts.

The challenge of predicting such variable outcomes from relatively sparse clinical and imaging features highlights the need for larger datasets and potentially more advanced modelling approaches in future studies. It also underscores the complexity of glioma progression and the multifactorial nature of treatment response and survival in this aggressive disease.

S2: Established prognostic factors in our cohort

Our analysis confirmed the prognostic significance of established molecular and demographic factors in grade 4 glioma.

Among the 187 patients with confirmed *IDH* status, *IDH* mutation was independently associated with substantially prolonged survival compared to IDH-wildtype tumors (median 28.9 months [IQR 27.3-33.9] versus 17.5 months [IQR 11.7-23.2], Supplementary Figure 1A. HR 0.46 [95% CI 0.25-0.86], p=0.014), although no longer significant after adjustment, probably due to the low number ow *IDH*-mutated in our cohort, Supplementary Figure 1A.

MGMT promoter methylation emerged as a strong positive prognostic indicator, with significantly improved overall survival in methylated versus unmethylated tumors (median 22.2 months [IQR 14.5-31.2] versus 16.7 months [IQR 11.5-22.1]; HR 1.80 [95% CI 1.28-2.53], p<0.001), Supplementary Figure 1B. MGMT methylation also conferred longer time to recurrence (10.5 months [IQR 6.6-18.3] versus 8.6 months [IQR 6.1-11.9]; HR 1.65 [95% CI 1.17-2.31], p=0.004).

Age at diagnosis demonstrated a consistent inverse relationship with survival outcomes with increased mortality risk (hazard ratio 1.03 per year [95% CI 1.01-1.04], p<0.001), and overall significantly improved survival for younger age quartiles, Supplementary Figure 1C.

We found no significant associations between extent of surgical resection and anatomical tumor location, indicating that resectability was relatively consistent across different brain regions in our cohort. The distribution of surgical approaches (gross total resection, subtotal resection, and biopsy) across various anatomical locations is detailed in Supplementary Figure 1D. Notable differences in surgical management are evident, with frontoparietal junction tumors receiving the highest rate of gross total resection (66.7%), while frontal lobe tumors had the lowest (31.2%). Conversely, frontal tumors had the highest proportion of subtotal resections (56.2%), while occipital tumors had the highest biopsy rate (27.3%). These patterns reflect the influence of anatomical accessibility and functional considerations on surgical decision-making for gliomas in different brain regions.

Within the IDH-wildtype cohort, we found consistent Cox Regression HR as in the full cohort, Supplementary Figure 1E.

Supplementary Figure 1

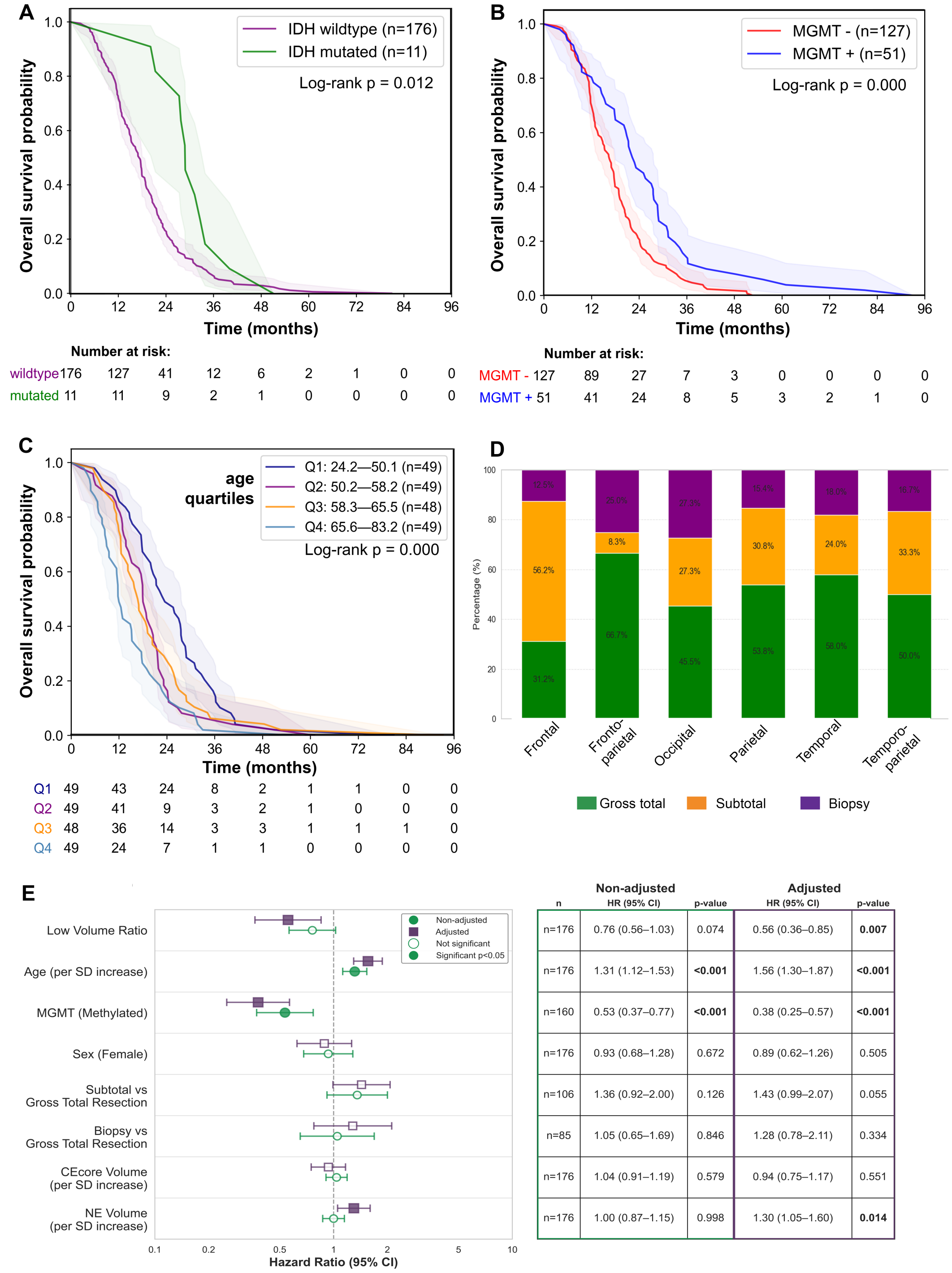

	n	Non-adjusted HR (95% CI)	p-value	Adjusted HR (95% CI)	p-value
Low Volume Ratio	n=176	0.76 (0.56–1.03)	0.074	0.56 (0.36–0.85)	**0.007**
Age (per SD increase)	n=176	1.31 (1.12–1.53)	**<0.001**	1.56 (1.30–1.87)	**<0.001**
MGMT (Methylated)	n=160	0.53 (0.37–0.77)	**<0.001**	0.38 (0.25–0.57)	**<0.001**
Sex (Female)	n=176	0.93 (0.68–1.28)	0.672	0.89 (0.62–1.26)	0.505
Subtotal vs Gross Total Resection	n=106	1.36 (0.92–2.00)	0.126	1.43 (0.99–2.07)	0.055
Biopsy vs Gross Total Resection	n=85	1.05 (0.65–1.69)	0.846	1.28 (0.78–2.11)	0.334
CEcore Volume (per SD increase)	n=176	1.04 (0.91–1.19)	0.579	0.94 (0.75–1.17)	0.551
NE Volume (per SD increase)	n=176	1.00 (0.87–1.15)	0.998	1.30 (1.05–1.60)	**0.014**

Supplementary Figure 1: Kaplan–Meier survival analyses stratified by established prognostic factors, distribution of surgical approaches by anatomical location, and multivariable Cox regression in the IDH-wildtype cohort. *A) Kaplan–Meier overall survival curves stratified by isocitrate dehydrogenase (IDH) mutation status: IDH-wildtype (n=176, purple) and IDH-mutated (n=11, green). Log-rank p=0.0120. Numbers at risk are shown below the curves. B) Kaplan–Meier overall survival curves stratified by O⁶-methylguanine-DNA methyltransferase (MGMT) promoter methylation status: unmethylated (MGMT−, n=127, red) and methylated (MGMT+, n=51, blue). Log-rank p<0.0001. Numbers at risk are shown below the curves. C) Kaplan–Meier overall survival curves stratified by quartiles of age at diagnosis: Q1 (24.2–50.1 years,*

n=49, dark blue), Q2 (50.2–58.2 years, n=49, purple), Q3 (58.3–65.5 years, n=48, orange), and Q4 (65.6–83.2 years, n=49, light blue). Log-rank p<0.0001. Numbers at risk are shown below the curves. D) Distribution of surgical interventions by primary tumor anatomical location, shown as a stacked bar chart. Each vertical bar represents the complete case distribution (100%) for a specific anatomical location (frontal, fronto-parietal, occipital, parietal, temporal, temporo-parietal), with color-coded segments indicating the relative frequency of each surgical approach: gross total resection (green), subtotal resection (orange), and biopsy (purple). Numerical percentages are overlaid on each segment. E) Multivariable Cox proportional hazards regression model for overall survival in the IDH-wildtype cohort (n=172). The forest plot displays hazard ratios with 95% confidence intervals for low CEcore/NE volume ratio (versus high), age (per standard deviation increase), MGMT methylation status, sex (female versus male), extent of resection (subtotal versus gross total resection; biopsy versus gross total resection), contrast-enhancing core (CEcore) volume (per standard deviation increase), and non-enhancing (NE) volume (per standard deviation increase). Non-adjusted estimates are shown as purple squares and adjusted estimates as green circles; filled symbols denote statistically significant associations (p<0.05) and open symbols non-significant associations. The vertical dashed line marks the null effect (hazard ratio = 1). The accompanying table reports sample sizes, hazard ratios with 95% confidence intervals, and p-values for both non-adjusted and adjusted models.

S3: Group sensitivity analysis

We performed comprehensive comparison of clinical, molecular, and volumetric characteristics between the low- and high-ratio groups (n=92 and n=103, respectively). The two groups did not differ significantly in age (median 58.3 vs. 58.2 years, p=0.9524), sex (p=0.5434), *MGMT* promoter methylation status (23.9% vs. 28.2% methylated, p=0.6194), or *IDH* mutation status (6.5% vs. 4.9% mutant, p=0.7568), Supplementary Table 2. Extent of resection did differ significantly between groups (p=0.0107). However, the pattern is not unidirectional: while the low-ratio group had a higher GTR-rate (39.1% vs. 34.0%), it also had a substantially higher biopsy rate (18.5% vs. 7.8%), whereas the high-ratio group had more subtotal resections (34.0% vs. 18.5%).

Supplementary Table 2:

Variable	Low ratio (≤0.324) n=92	High ratio (>0.324) n=103	Test	p
**Age at diagnosis (years)**	58.3 (49.8–65.3)	58.2 (50.4–65.4)	Mann–Whitney U	0.9524
**Sex**				
Male	59 (64.1%)	71 (68.9%)	Fisher's exact	0.5434
Female	33 (35.9%)	32 (31.1%)		
**MGMT promoter methylation**				
Unmethylated	61 (66.3%)	66 (64.1%)	Fisher's exact	0.6194
Methylated	22 (23.9%)	29 (28.2%)		
Missing	9 (9.8%)	8 (7.8%)		
**IDH mutation status**				
Wildtype	80 (87.0%)	96 (93.2%)	Fisher's exact	0.7568
Mutant	6 (6.5%)	5 (4.9%)		
Missing	6 (6.5%)	2 (1.9%)		
**Extent of resection**			Chi-square	0.0107*
Gross total	36 (51.4%[1])	35 (44.9%[1])	Fisher's exact	0.5101
Subtotal	17 (24.3%[1])	35 (44.9%[1])	Fisher's exact	0.0100*
Biopsy	17 (24.3%[1])	8 (10.3%[1])	Fisher's exact	0.0283*
Missing	22	25		
**Tumor volume**				
CEcore volume (cm³)	9.9 (3.0–25.2)	33.5 (17.3–58.2)	Mann–Whitney U	<0.0001***
NE volume (cm³)	86.9 (35.7–126.8)	46.9 (23.7–94.2)	Mann–Whitney U	0.0041**
**Overall survival (months)**	19.0 (13.5–26.9)	17.0 (11.4–23.0)	Mann–Whitney U	0.0281*

[1]Of cases with known EOR

Supplementary Table 2. Comparison of clinical, molecular, and volumetric characteristics between low and high CEcore/NE volume ratio groups*. Baseline characteristics are presented for patients stratified by the contrast-enhancing core to non-enhancing (CEcore/NE) volume ratio at the binary threshold of 0.324 (low ratio, ≤0.324, n=92; high ratio, >0.324, n=103). Continuous variables are reported as median (interquartile range, IQR) and compared using the Mann–Whitney U test. Categorical variables are reported as n (%) and compared using Fisher's exact test, except for extent of resection (EOR), which was tested overall using the chi-square test with subsequent pairwise Fisher's exact tests for each surgical category (gross total resection, subtotal resection, biopsy). Missing values are listed separately and were excluded from the corresponding statistical tests. For EOR, percentages are calculated from cases with known resection status (denoted by the superscript 1), with missing cases shown as raw counts. Abbreviations: MGMT, O^6-methylguanine-DNA methyltransferase; IDH, isocitrate dehydrogenase; CEcore, contrast-enhancing core; NE, non-enhancing; EOR, extent of resection. Statistical significance is denoted by asterisks: *p<0.05, **p<0.01, ***p<0.001.*

S4: CEcore/NE volume ratio: full cohort vs *IDH* wildtype cohort

Supplementary figure 2

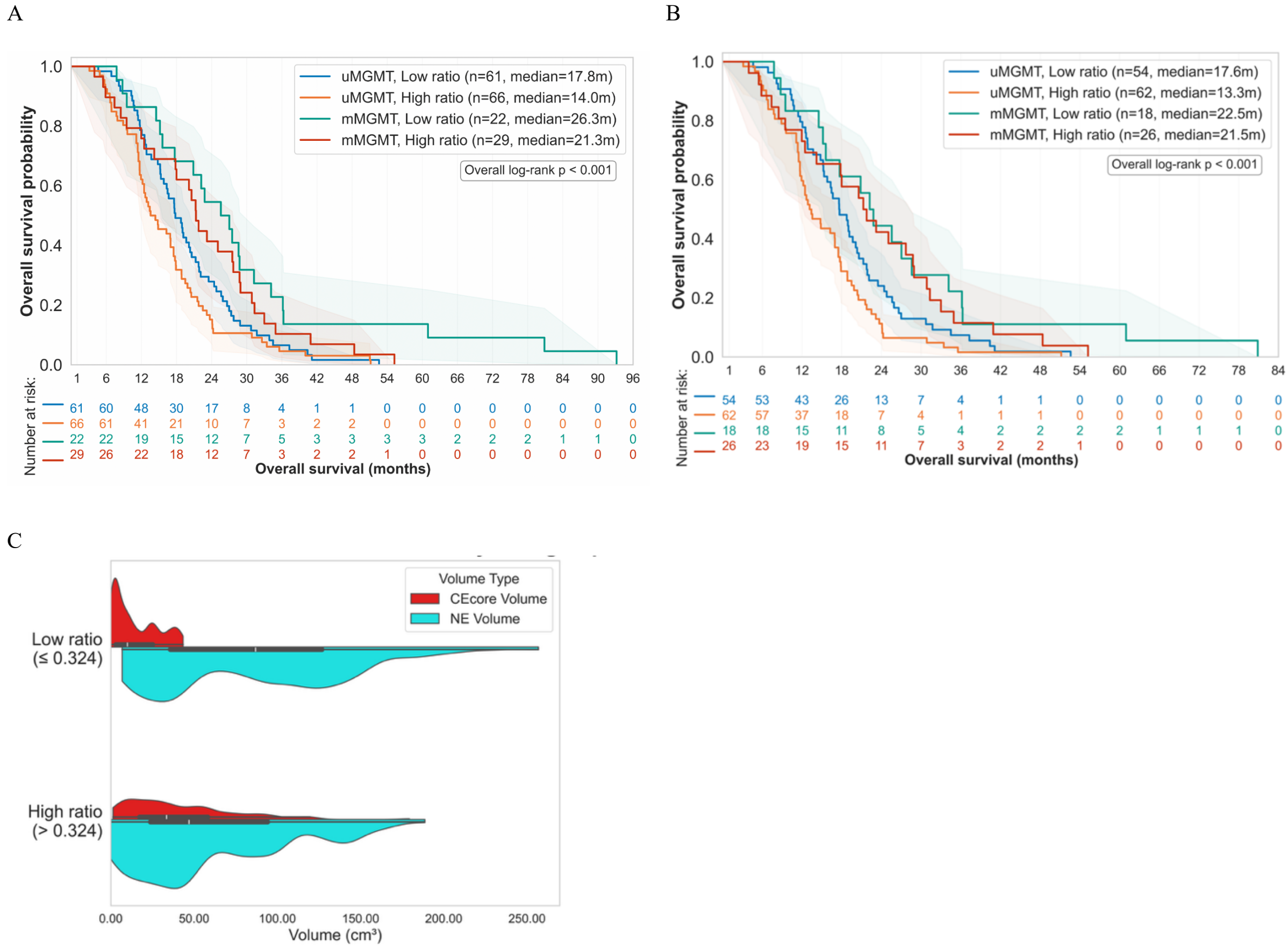

Supplementary Figure 2: Survival stratified jointly by MGMT methylation status and CEcore/NE volume ratio in the full cohort and the IDH-wildtype subset, and distribution of tumor compartment volumes across ratio groups. *A) Kaplan–Meier overall survival curves in the full cohort (n=178 with known MGMT status), stratified jointly by O⁶-methylguanine-DNA methyltransferase (MGMT) promoter methylation status and the contrast-enhancing core to non-enhancing (CEcore/NE) volume ratio at the binary threshold of 0.324: unmethylated MGMT (uMGMT) with low ratio (≤0.324; n=61, median overall survival 17.8 months, blue), uMGMT with high ratio (>0.324; n=66, median 14.0 months, orange), methylated MGMT (mMGMT) with low ratio (n=22, median 26.3 months, teal), and mMGMT with high ratio (n=29, median 21.3 months, red). Overall log-rank p<0.0001. Shaded bands indicate 95% confidence intervals. Numbers at risk for each group are shown below the curves in matching colors. B) Kaplan–Meier overall survival curves restricted to the isocitrate dehydrogenase wildtype (IDH-wildtype) subset (n=160 with known MGMT status), stratified jointly by MGMT methylation status and the CEcore/NE volume ratio: uMGMT with low ratio (n=54, median overall survival 17.6 months, blue), uMGMT with high ratio (n=62, median 13.3 months, orange), mMGMT with low ratio (n=18, median 22.5 months, teal), and mMGMT with high ratio (n=26, median 21.5 months, red). Overall log-rank p<0.0001. Shaded bands indicate 95% confidence intervals. Numbers at risk for each group are shown below the curves in matching colors. C) Density (ridge) plot showing the distribution of CEcore tumor volume (red) and NE tumor volume (cyan) in cm³, separately for patients with a low CEcore/NE volume ratio (≤0.324, top) and a high CEcore/NE volume ratio (>0.324, bottom). Horizontal lines beneath each density indicate the interquartile range.*

S5: Cohort sensitivity analysis

As the study cohort comprises three independent datasets (historical controls, n=97; NCT03643549 trial, n=57; UPENN-GBM, n=41) with known inter-cohort differences in MGMT status (by trial design) and age (Supplementary Table 3), we assessed whether cohort membership confounded the prognostic value of the CEcore/NE volume ratio.

Supplementary Table 3: Cox regression sensitivity analysis for overall survival comparing primary model with cohort-adjusted model

Variable	*Contemporary controls (n=97)*	*NCT03643549 trial (n=57)*	*UPENN-GBM (n=41)*	*p-value*
Age *(years)*	61.0 [52.3–67.4]	54.0 [47.7–61.1]	57.9 [52.5–64.2]	0.0061
Sex				0.7958
Female	34 (35.1%)	17 (29.8%)	14 (34.1%)	
Male	63 (64.9%)	40 (70.2%),	27 (65.9%),	
MGMT				<0.0001
Unmethylated	53 (54.6%)	57 (100.0%)	17 (70.8%),	
Methylated	44 (45.4%)	0 (0.0%)	7 (29.2%)	
IDH				0.0936
Wildtype	85 (94.4%),	51 (89.5%)	40 (100.0%)	
Mutant	5 (5.6%)	6 (10.5%)	0 (0.0%)	
EOR				0.2920
GTR	45 (46.4%)	26 (51%[1])		
STR	38 (39.2%),	14 (27%[1]),		
Biopsy	14 (14.4%)	11 (21%[1]),		
Unknown		6	41 (100.0%)	
Time to progression (months)	7.7 [6.1–12.5]	10.0 [7.8–13.1]	8.6 [4.9–13.8]	0.2368
Overall survival (months)	17.0 [10.3–25.6]	19.0 [13.4–24.3]	18.6 [12.4–24.4]	0.1579
HD95 (mm)	19.2 [11.4–32.3]	20.9 [11.6–36.2]	16.1 [7.9–26.6]	0.1904

[1] Of known EOR-status

Supplementary Table 3. Baseline clinical, molecular, treatment, and outcome characteristics across the three constituent cohorts. *Patient and tumor characteristics are presented separately for the three cohorts comprising the study population: contemporary controls (n=97), the NCT03643549 trial (n=57), and UPENN-GBM (n=41). Continuous variables are reported as median [interquartile range, IQR] and compared across cohorts using the Kruskal–Wallis test. Categorical variables are reported as n (%) and compared using the chi-square test. For extent of resection (EOR), percentages in the NCT03643549 trial column are calculated from cases with known resection status (denoted by the superscript 1); the UPENN-GBM cohort had no available EOR data and is shown as 100% unknown. Time to progression and overall survival are reported in months. Abbreviations: MGMT, O^6-methylguanine-DNA methyltransferase; IDH, isocitrate dehydrogenase; EOR, extent of resection; GTR, gross total resection; STR, subtotal resection; HD95, 95th percentile Hausdorff distance between primary and recurrent contrast-enhancing tumor cores.*

We also fitted a sensitivity Cox regression model identical to the primary adjusted model (Figure 2C), with two additional cohort variables (NCT03643549 trial and UPENN-GBM, with historical controls as reference). The CEcore/NE volume ratio remained independently prognostic (HR=0.59, 95% CI: 0.39–0.89, p=0.0126), consistent with the primary model (HR=0.56, 95% CI: 0.37–0.84, p=0.0055). All other covariates maintained consistent direction and significance patterns (Supplementary Table 3). The condition number remained at 5.6, indicating no multicollinearity. The NCT03643549 trial cohort was significant (HR=0.64, 95% CI: 0.44–0.94, p=0.0236), likely reflecting a trial participation effect, whereas the UPENN-GBM cohort showed a borderline non-significant effect (HR=0.61, 95% CI: 0.37–1.01, p=0.0546). Model comparison showed marginal improvement in concordance (0.694 vs 0.700) and AIC (1389.5 vs 1386.5) with the addition of cohort, while a likelihood ratio test was significant (chi-squared=7.02, df=2, p=0.0300), indicating that cohort membership captures residual variance - most likely attributable to the trial participation effect - but does not confound the biomarker associations. These results confirm that the independent prognostic value of the CEcore/NE volume ratio is not attributable to inter-cohort differences.

S6: HD95 in relation to EOR and anatomical location and sex

Overall, neither EOR, anatomical location nor sex showed any correlation with HD95 measurements. Using ANCOVA, we fitted separate models for each tumor compartment (CEcore and NE), with HD95 as the dependent variable, anatomical location as a categorical factor (temporal lobe as reference, n = 66), and EOR as a three-level categorical covariate (GTR, n = 71; STR, n = 52; biopsy, n = 25). Type II sums of squares were used to test each factor after adjustment for the other. Neither anatomical location nor EOR significantly predicted CEcore HD95 (location: F = 0.41, p = 0.8939; EOR: F = 0.05, p = 0.9542) or NE HD95 (location: F = 0.65, p = 0.7106; EOR: F = 0.45, p = 0.6375). In a sensitivity analysis additionally adjusting for MGMT promoter methylation status, IDH mutation status, age, and sex (n = 141), results remained consistent (all p > 0.49).

S7: Cox regression of overall survival by primary tumor location IDH wildtype cohort

Cox regression analysis demonstrated that midline/bilateral tumors had significantly worse overall survival prognosis, Supplementary Figure 3.

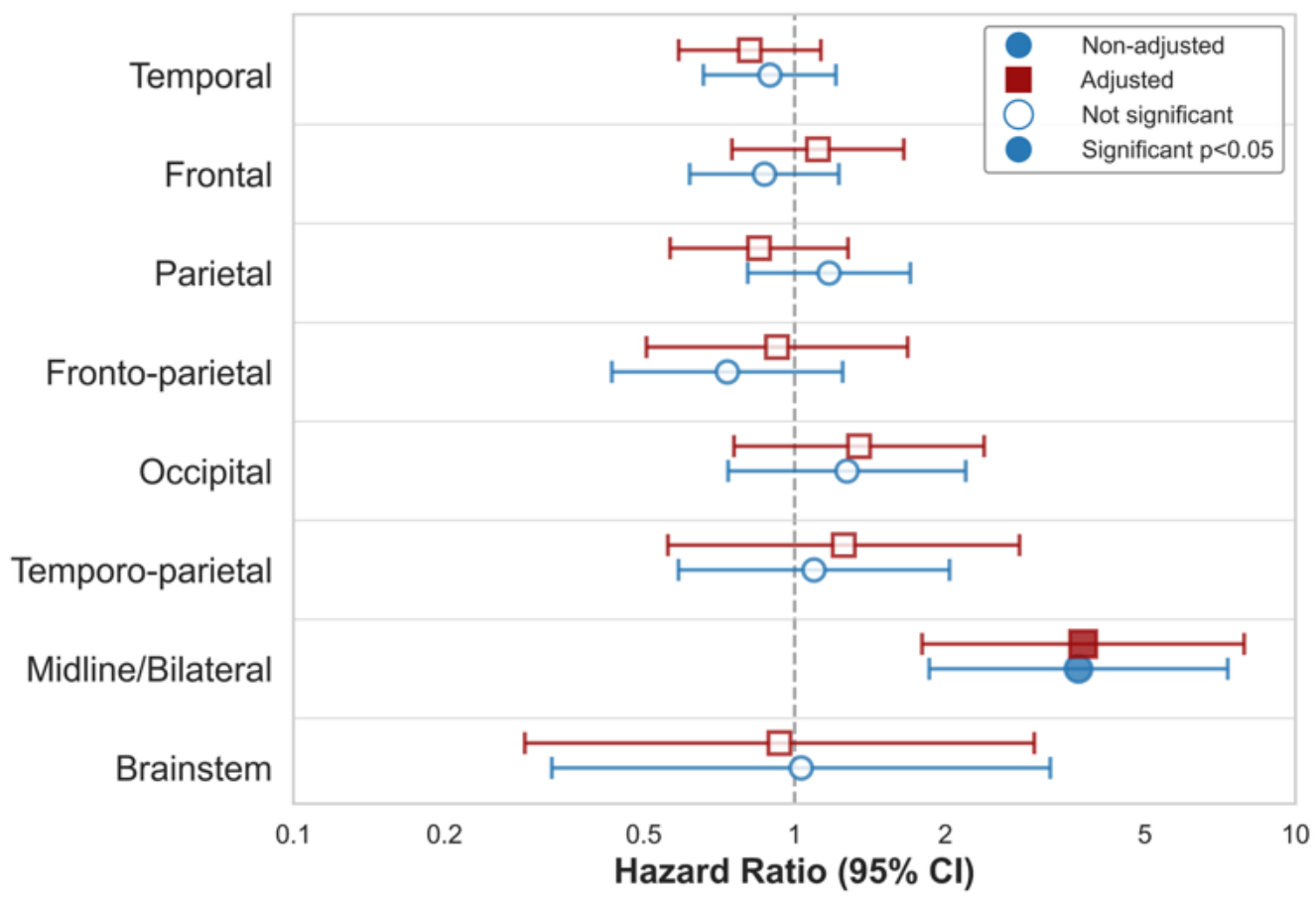

Non-adjusted HR (95% CI)	p-value	Adjusted HR (95% CI)	p-value
0.89 (0.66–1.21)	0.464	0.81 (0.59–1.13)	0.217
0.87 (0.62–1.23)	0.428	1.11 (0.75–1.65)	0.594
1.17 (0.81–1.70)	0.406	0.85 (0.56–1.28)	0.436
0.73 (0.43–1.25)	0.254	0.92 (0.51–1.68)	0.794
1.27 (0.74–2.20)	0.387	1.35 (0.76–2.39)	0.311
1.09 (0.59–2.04)	0.779	1.25 (0.56–2.81)	0.583
3.69 (1.86–7.32)	**<0.001**	3.77 (1.80–7.89)	**<0.001**
1.03 (0.33–3.24)	0.959	0.93 (0.29–3.01)	0.909

Supplementary Figure 3: Cox proportional hazards regression of overall survival in IDH-wt patients by primary tumor anatomical location. *Forest plot showing hazard ratios with 95% confidence intervals for overall survival, comparing each primary tumor location separately against all other locations combined (one-versus-rest analysis): temporal, frontal, parietal, fronto-parietal, occipital, temporo-parietal, midline/bilateral, and brainstem. Non-adjusted estimates are shown as blue circles and adjusted estimates as red squares; open symbols denote non-significant associations and filled symbols denote statistically significant associations (p<0.05). Horizontal lines indicate 95% confidence intervals and the vertical dashed line marks the null effect (hazard ratio = 1). Adjusted models were restricted to patients with complete molecular data (n=168) and included O^6-methylguanine-DNA methyltransferase (MGMT) promoter methylation status, isocitrate dehydrogenase (IDH) mutation status, sex, age at diagnosis, and extent of surgical resection as covariates. The accompanying table reports per-model sample*

sizes, hazard ratios with 95% confidence intervals, and p-values for both non-adjusted and adjusted models. Midline/bilateral tumors were associated with significantly worse overall survival in both non-adjusted (HR=3.69, 95% CI 1.86–7.32, p<0.0001) and adjusted (HR=3.77, 95% CI 1.80–7.89, p<0.0001) analyses.

S8: Co-registration across time-points

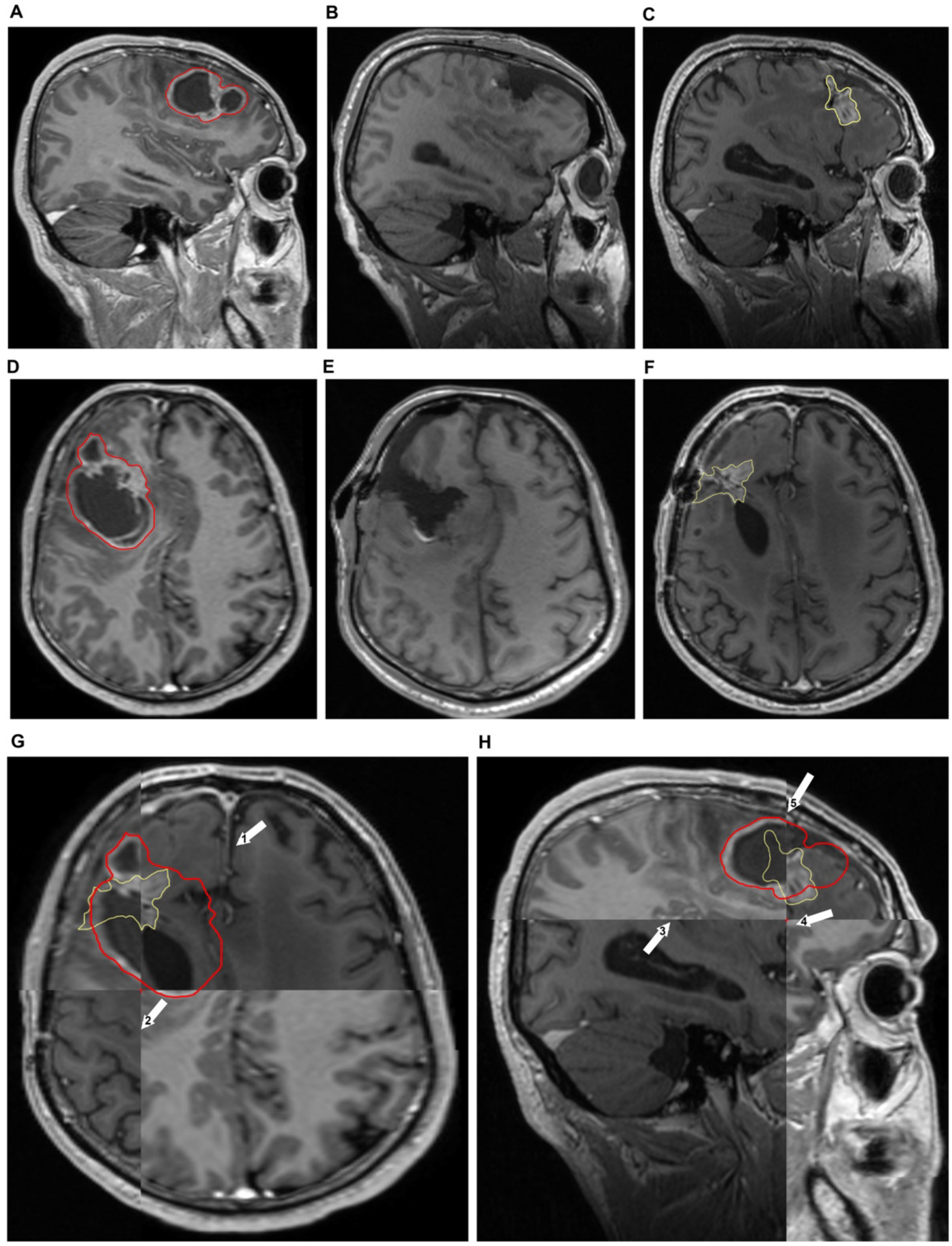

Supplementary Figure 4: **Representative case illustrating the deep learning–based segmentation and deformable co-registration workflow used to quantify spatial *recurrence patterns.***

A–C) Sagittal T1-weighted contrast-enhanced MRI of a representative grade 4 glioma case at three timepoints: A) at diagnosis, with the autosegmented contrast-enhancing core (CEcore) outlined in red; B) postoperative scan; C) at established recurrence 16.1 months after diagnosis, with the autosegmented recurrent CEcore outlined in yellow.

D–F) Corresponding axial T1-weighted contrast-enhanced MRI of the same case at the matching timepoints: D) at diagnosis (primary CEcore outlined in red); E) postoperative scan; F) at recurrence (recurrent CEcore outlined in yellow). G–H) Quality control of the deformable co-registration between diagnostic and recurrence scans, displayed as a checkerboard overlay in which alternating tiles are drawn from the two registered image volumes; concordance is judged by the continuity of anatomical structures across tile boundaries. Both segmentations are overlaid (primary CEcore in red, recurrent CEcore in yellow). G) Axial checkerboard view; H) sagittal checkerboard view. Numbered white arrows indicate landmarks evaluated for alignment: arrow 1, the falx cerebri; arrows 2–4, the cortical surface and major sulci in the area peripheral to the tumor; arrow 5, the dura. Additional landmarks evaluated but not depicted in this figure include the ventricular system, the tentorium cerebelli, and the brainstem.

S9: Anatomical tumor trajectory

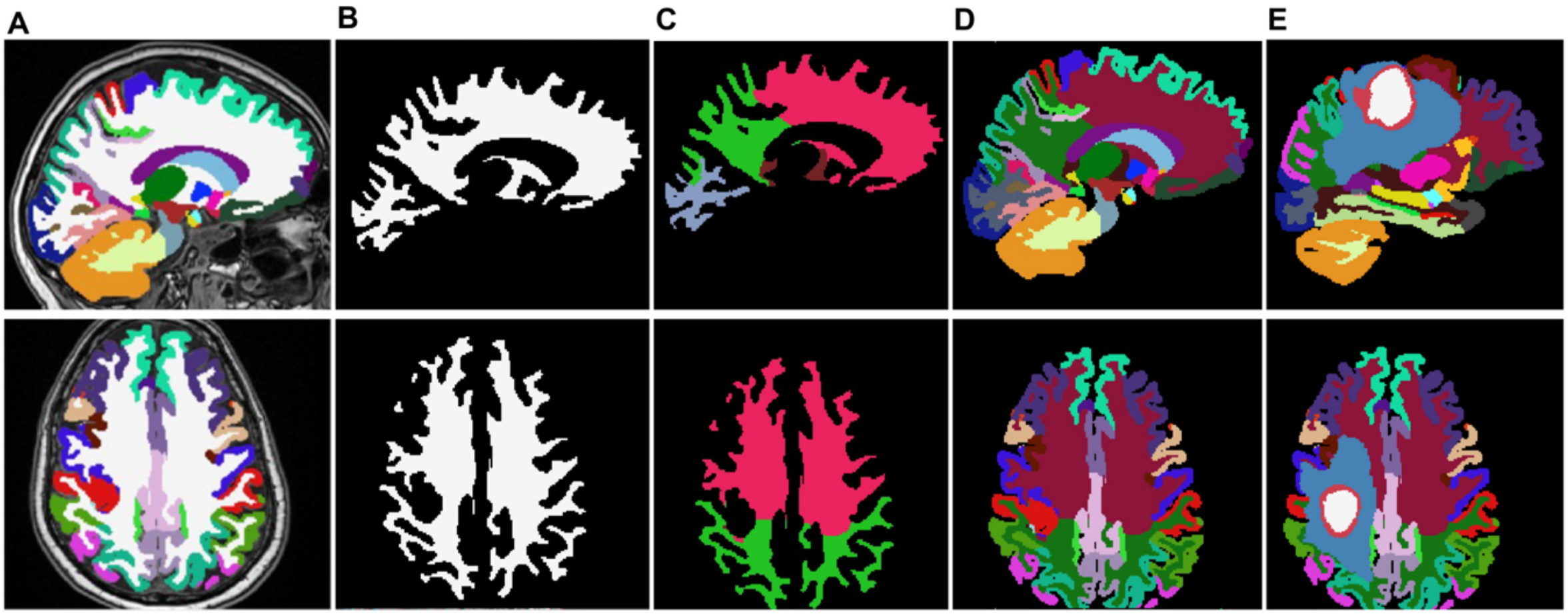

Supplementary Figure 5: Anatomical brain parcellation and lobar classification pipeline used to localize tumor compartments. *Sequential outputs of the anatomical processing pipeline are shown in sagittal view (top row, panels A–E) and matching axial view (bottom row) for a representative case. AF) SynthSR-reconstructed high-resolution T1-weighted image overlaid with the SynthSeg+ anatomical parcellation, which generates 101 distinct anatomical labels comprising 68 cortical parcellations across both hemispheres, 28 subcortical structures, and 5 central structures. Cortical regions are color-coded by parcellation label. B) Binary hemispheric white matter mask extracted from the SynthSeg+ output. SynthSeg+ generates white matter segmentations only as unified left and right hemispheric volumes, without further lobar differentiation, shown here in white on a black background. C) Lobular classification of hemispheric white matter using a K-nearest neighbors algorithm (K=3), which assigns each white matter voxel to a specific lobar region (frontal, parietal, temporal, or occipital) based on its spatial proximity to the corresponding groups of cortical parcellations from the SynthSeg+ output. Lobar regions are color-coded, with cortical parcellations retained for anatomical reference. D) the refined lobular white-matter segments from C in context with cortical segmentations in A. E) Final composite output combining the anatomical classification with the contrast-enhancing tumor compartment (red), the necrotic core (white) and the non-enhancing tumour (blue). This anatomical framework forms the basis for the tumor overlap analysis used to classify tumors at a specific lobe, either as as*

single-lobe dominant, junction, or bilateral, and to track anatomical migration patterns between primary diagnosis and recurrence (see Methods, Anatomical brain parcellation and tumor localization).

S10: Derivation of the CEcore/NE ratio threshold

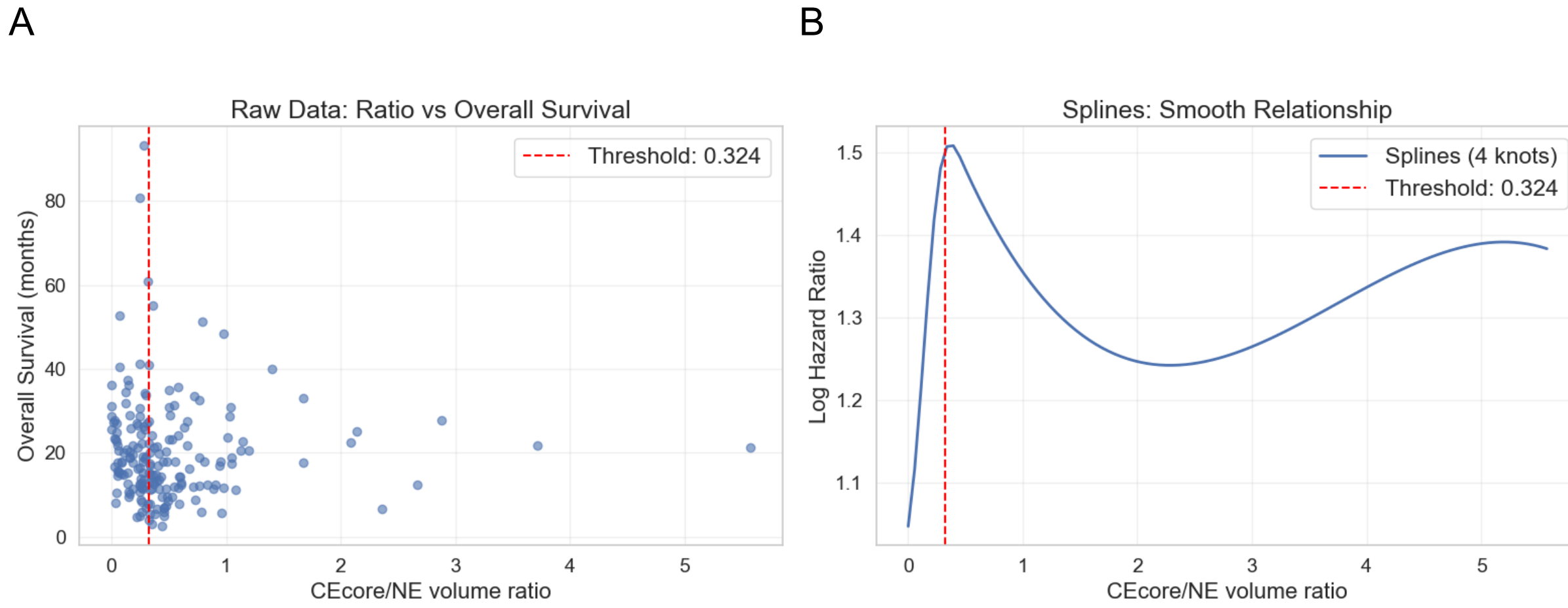

Supplementary Figure 6: Threshold determination for the CEcore/NE volume ratio: distribution of overall survival and restricted cubic spline analysis of the log hazard ratio. *A) Scatter plot of overall survival (months) against the contrast-enhancing core to non-enhancing (CEcore/NE) volume ratio (n=192 patients), with each blue point representing one patient. The vertical red dashed line indicates the binary threshold of 0.324. B) Best-performing restricted cubic spline curve (4 knots, selected from 3–8 knots tested) showing the log hazard ratio as a function of the CEcore/NE volume ratio (solid blue line). The vertical red dashed line marks the binary threshold of 0.324*
